# Scalable Purification of Iron Oxide Nanoparticles for Organ Cryopreservation and Transplantation

**DOI:** 10.1002/smll.202504910

**Published:** 2025-09-24

**Authors:** Onyinyechukwu Justina Oziri, Joseph Sushil Rao, Cameron Scheithauer, Zonghu Han, Saurin Kantesaria, Diane Tobolt, Haopu Liang, Michael L Etheridge, Yadong Yin, Erik B Finger, John C Bischof

**Affiliations:** ^1^ Department of Mechanical Engineering University of Minnesota Minneapolis MN 55455 USA; ^2^ Department of Surgery University of Minnesota Minneapolis MN 55455 USA; ^3^ Center for Magnetic Resonance Research and Department of Radiology University of Minnesota Medical School Minneapolis MN 55455 USA; ^4^ Department of Chemistry University of California Riverside CA 92521 USA; ^5^ Department of Biomedical Engineering University of Minnesota Minneapolis MN 55455 USA; ^6^ Institute for Engineering in Medicine University of Minnesota Minneapolis MN 55455 USA

**Keywords:** cryopreservation, iron oxide nanoparticles, nanowarming, tangential flow filtration, ultracentrifugation

## Abstract

Our group recently reported the successful transplant of cryopreserved rat kidneys that independently supported the life of recipient rats, marking a key step toward human‐scale organ cryopreservation. In vitrification, organs are cooled to cryogenic temperatures and transformed into a glassy state, allowing indefinite storage. However, rewarming without ice formation or thermal stress‐induced cracking remains a challenge. To overcome this, a technique called “nanowarming,” is pioneered in which iron oxide nanoparticles (IONPs) are perfused into the organ's vasculature in combination with cryoprotective agents (CPAs). When rewarmed using a radiofrequency coil, the nanoparticles generate heat, enabling rapid and uniform warming. This method is scalable to large volumes because RF frequencies penetrate with minimal attenuation. While nanowarming is demonstrated with rat organs, clinical‐scale organs will require large quantities of IONPs that must remain stable in CPAs and provide effective heating. To scale up IONP purification, tangential flow filtration (TFF) is used, which achieved the purification of 0.72 g IONPs in 2.5 h. This method reduces IONP loss and residual content in the organ, ensuring effective heating while maintaining stability. Finally, the method by successfully transplanting cryopreserved and nanowarmed rat kidneys is validated.

## Graphical abstract

Scalable purification of silica‐coated iron oxide nanoparticles (sIONP) enables organ‐scale nanowarming and cryopreservation. Tangential flow filtration (TFF) offers a rapid and scalable alternative to conventional ultracentrifugation, completing purification in 2.5 h. The resulting sIONP exhibits excellent stability in cryoprotective agents, sufficient heating performance, biocompatibility, reduced residual deposition, and supports successful kidney transplantation.

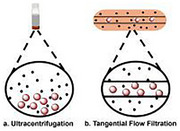

## 1 Introduction

Preserving whole organs by cryopreservation to create organ banks and facilitate organ storage and logistics has been a long‐time goal in facilitating lifesaving organ transplants.^[^ ^1^ ^]^ Organ transplantation remains the sole treatment option in many cases of end‐stage organ disease and failure. Although organ donation has increased in recent years, the short storage time clinically allowable for organs means that a significant portion of organs are never used.^[^ ^1^, ^2^ ^]^ Cryopreservation and indefinite storage of organs by vitrification could provide access to more organs, allow time to induce immune intolerance induction, and ultimately improve donor/recipient matching and life expectancy of both organs and patients.^[^ ^3^ ^]^ Cryopreservation is often cited as beginning with the first successful preservation of fowl sperm in 1949.^[^ ^4^ ^]^ Since then, researchers have successfully cryopreserved many different cells, tissues, and, more recently, organs.^[^ ^5^, ^6^, ^7^, ^8^, ^9^ ^]^ For instance, our group recently demonstrated cryopreservation, storage (up to 100 days), and rewarming of a rat kidney followed by transplantation, which independently supported the life of the recipient rat.^[^ ^3^ ^]^ Other work also shows encouraging data on the cryopreservation of a rabbit kidney.^[^ ^9^ ^]^

Successful organ cryopreservation requires two main processes: vitrification and rewarming. Since the 1980s, vitrification has used a high concentration of cryoprotectant agents (CPAs) perfused in and around the organ so that it turns into a glassy state during cooling to cryogenic temperature to avoid ice formation and its associated injury to the organs. Successful vitrification is achieved when the cooling rate exceeds the critical cooling rate (CCR) of the cryoprotective agent (CPA), typically in the 1–10 °C min^−1^ range, and is achievable with existing technology. Unfortunately, the cooling step seeds the system with many ice nuclei that require a much higher warming rate, typically 50–100 °C min^−1^,^[^ ^10^ ^]^ to avoid crystal growth upon warming. This is much harder, if not impossible, to achieve in bulk systems by boundary warming (convection) approaches. Faster warming on the boundary also introduces thermal gradients in the sample, which can drive thermal stress and cracking.^[^ ^11^, ^12^, ^13^ ^]^ To address this, our group developed a volumetric and scalable technique called nanowarming, in which iron oxide nanoparticles (IONPs) are introduced into the organ vasculature along with CPA solutions before vitrification.^[^ ^14^, ^15^, ^16^, ^17^, ^18^ ^]^ The organs can be rewarmed on demand by applying an alternating magnetic field generated by a radiofrequency coil. It heats the nanoparticles within the organ vasculature and its surroundings, achieving rapid and uniform warming and preventing ice crystallization and cracking. Notably, the IONP remains in the vasculature throughout the process, so they are washed out of the organ during CPA unloading before transplant. Nanowarming requires IONPs in the CPA to be highly stable, biocompatible, capable of high heating, cheap, and scalable via both synthetic and purification methods.^[^ ^14^, ^19^, ^20^ ^]^

**Figure 1:**
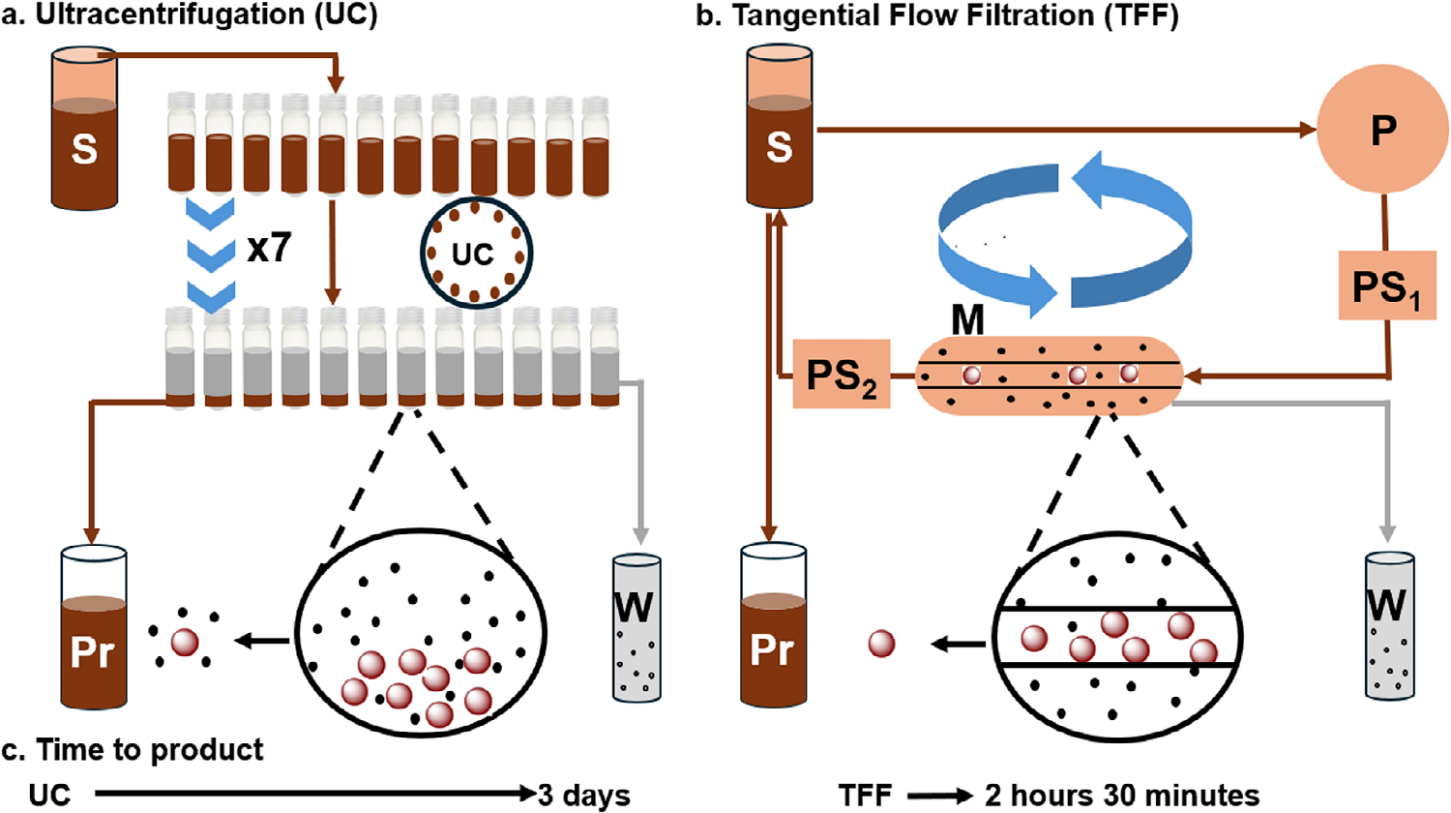
Schematic representation of the purification workflows using tangential flow filtration (TFF) and ultracentrifugation (UC): a) UC workflow representing a batch purification process; b) TFF workflow illustrating a semi‐continuous purification process; c) Comparison of total processing time required to purify 0.72 g of iron using TFF and UC methods. In the schematic: S indicates sIONP in water; P, the pump; PS_1_ and PS_2_, the first and second pressure sensors; M, the membrane filter; Pr, the purified product; and W, the waste.

In the past, we have demonstrated successful nanowarming using silica‐coated iron oxide nanoparticles (sIONP) purified by ultracentrifugation (UC, **Figure** **1**b).^[^ ^20^ ^]^ Ultracentrifugation is a common method for nanoparticle purification that utilizes centrifugal force to separate the final product from undesired materials (e.g., unreacted components).^[^ ^21^ ^]^ Upon centrifugation, the g‐force generated leads to caking and densely packed pellets, requiring laborious efforts and methods to redisperse the nanoparticles. Furthermore, centrifugation of up to 0.72 g (one‐half of our current 1.44g limit for lab scale IONP synthesis, but can be increased further through industrial processes) results in, 2 L of ethanol usage, 150–250 mg sIONP loss, a labor‐intensive process (3 days of manual processing), and batch‐to‐batch variability.^[^ ^22^, ^23^ ^]^ Ultracentrifugation (UC) introduces several operator‐dependent variables, including manual steps such as dispersion, decanting, solvent exchange, and pellet resuspension. Additionally, process parameters such as centrifugation speed, time, and rotor type can vary between batches and across operators, leading to significant variability. Batch‐to‐batch variation in UC is illustrated in Table S3 (Supporting Information), which shows differences in the sizes of IONPs produced. Scaling UC requires multiple parallel runs and increased labor, further compounding inconsistencies. In contrast, tangential flow filtration (TFF) is a closed system that enables continuous and reproducible purification. It operates under fixed, controllable parameters (e.g., transmembrane pressure, flow rate, membrane cut‐off), and scaling is achieved simply by increasing membrane surface area or volume processed—without altering core conditions. As a result, TFF minimizes human error and provides significantly greater batch‐to‐batch consistency, especially when scaled to clinically relevant production levels. Furthermore, the fixed size of the centrifugal tubes (26×77 mm size, 29.9 mL capacity) limits the scalability of ultracentrifugation above 1.44 g of sIONP so that these issues will increase proportionally with attempts at larger scale production. These limitations are a significant concern when translating nanowarming to clinical‐scale organs.

To address these obstacles, we demonstrate tangential flow filtration (TFF) for sIONP purification. TFF is a method that employs tangential flow recirculation of sIONP across a selectively permeable membrane under pressure (Figure 1b). Notably, the TFF process is faster, less labor‐intensive, more scalable (e.g., it can easily increase from 0.72 to 10 g with the acquisition of process‐scale instruments), and more clinically translatable (it is already utilized in biopharmaceutical manufacturing) than other methods. Herein, we developed and optimized TFF parameters to enable effective purification of 0.72 g of IONP in 2 h and 30 min without the 2 L of ethanol required for UC. This protocol resulted in stability in CPAs over 7 months, biocompatibility with human dermal fibroblasts (HDF), reduced nanoparticle residue after transplantation, and efficient heating (SAR value = 360.3 ± 25.53 W g^−1^ Fe). Finally, the TFF‐produced IONP were used to successfully demonstrate the transplantation of cryopreserved and nanowarmed rat kidneys in recipient rats.

## 2 Results and Discussion

### 2.1 Overview of IONP Purification

The historical development of IONPs through various purification methods for nanowarming is shown in **Table** **1**. These IONPs have been applied across multiple cells, tissue, and organ systems. Except for TFF, none of the purification methods simultaneously demonstrated stability or scalability, both of which are necessary for working with clinical‐scale organs.

**Table 1:** Different magnetic nanoparticles, their stability in CPAs, purification methods used, and potential for scalability.

Nanoparticles	Coating	Purification	CPA stability	Scalability	Refs.
EMG 308 (Ferrotec, USA)	Proprietary	Proprietary	No	Yes	[24]
Mesoporous silica IONPS	silica and PEG	Centrifugation	Yes	No	[25]
SPION	PEG‐silane	Dialysis and magnetic separation	Yes	No	[26]
phosphonate‐linked PEG IONPs	mPEG	Magnetic separation	Yes	No	[19]
Silica coated IONP	Silica, PVP and mPEG	Ultracentrifugation	Yes	No^a)^	[20]
IONP nanoclusters	Resorcinol‐formaldehyde resin (RFR)	Centrifugation	Yes	No	[27]
Silica‐coated IONP	Silica, PVP and mPEG	TFF	Yes	Yes^b)^	This study

^a)^ Single batch gram quantity batches (0.72 g IONPs in 3 days)

^b)^ Semi‐continuous process (0.72 g IONP in 2 h 30 mins))

The first demonstration of nanowarming was performed by Etheridge et al.,^[^ ^24^ ^]^ using a CPA solution with EMG 308, a surfactant‐coated IONP core that is commercially available and inexpensive. Unfortunately, EMG308 does not offer long‐term stability in CPA (crashes within minutes in VS55), thereby limiting its use in nanowarming,^[^ ^24^ ^]^ but it was effective for initial demonstrations of the approach. To improve stability in CPA, EMG 308 was coated with mesoporous silica IONP (msIONP) and achieved robust colloidal stability in CPA with a biological demonstration of nanowarming.^[^ ^14^ ^]^ However, the synthetic process, treatment, and purification for msIONP takes several days and requires elevated temperatures (50°C–90 °C), cetyltrimethylammonium bromide (CTAB) removal, and a laborious process.^[^ ^25^ ^]^ In other efforts, Chiu‐Lam et al. produced sIONP, which remained stable for 28 days in CPA but required purification through dialysis to eliminate excess PEG silane, followed by additional purification via magnetic separation, which is labor‐intensive, time‐consuming, and challenges scalability.^[^ ^26^ ^]^ Gao et al. removed the need for CTAB and developed a scalable silica‐coated EMG synthesis in a 4 L reaction vessel (1.44 g), which proved stable in CPA. However, the ultracentrifugation purification method requires several days to complete, thus restricting its scalability for clinical nanowarming practices.^[^ ^20^ ^]^ To improve scalability and increase the concentration of IONP in CPA, Pasek‐Allen et al. (2022) linked phosphonate‐linked PEG directly to IONPs without silica, but stability in CPA was limited to 7 days.^[^ ^19^ ^]^ Ye et al. engineered magnetic nanoclusters for effective nanowarming, achieving 21 days of stability in CPA, although purification was performed through centrifugation.^[^ ^27^ ^]^

Other researchers have reported synthesizing and purifying IONPs but have not provided specific CPA stability and scalability data. For instance, Liu et al.^[^ ^28^ ^]^ and Tian et al.^[^ ^29^ ^]^ utilized chemical co‐precipitation for IONP synthesis and magnetic separation for purification. Still, they did not present data on IONP stability in CPAs. Zhan et al. nanowarmed an entire vitrified rat kidney with IONP nanoparticles coated with carboxylic functionality. Yet, they did not provide information on the stability of IONPs in CPAs or the uniform distribution of particles in the kidney.^[^ ^30^ ^]^

The conventional ultracentrifugation method for sIONP purification presents significant scalability limitations. This arises from the use of Beckman Coulter 50. 2 Ti rotor and centrifuge tubes with dimensions of 26×77 mm and a maximum capacity of 29.9 mL. Additional challenges include the labor‐intensive nature of the process, difficulty in redispersing the IONP, the requirement of 2 L of ethanol, and a total purification time of 3 days to process for 0.72 g of IONP, a loss of 150–250 mg of IONP, and the presence of brown residues in kidneys after washout that may be associated with IONP. While some of these values may vary depending on the specific rotor and tube size used, it is important to note that our protocol already employs one of the largest high g force rotor and tube configurations commercially available. So, the scalability of the ultracentrifugation method beyond what is demonstrated here is limited.

In contrast, for the same amount of iron (0.72 g), tangential flow filtration (TFF), a dynamic filtration process for IONP purification, requires only 2 h and 30 min. This method allows IONPs to flow tangentially to the membrane under pressure, eliminates the need for redispersal, and does not require placement in size‐limited tubes. Furthermore, TFF results in no loss of IONP, facilitates easy post‐purification dispersion, and reduces the brown residues observed in histology after washout from the kidney, as shown. By understanding the advantages of the TFF purification process over ultracentrifugation, we can directly compare the use of IONP from each purification method for nanowarming.

Comparison of physical and biological assessments of sIONP, such as DLS size, zeta potential, TEM, viability, vitrifiability, perfusion pressure, flow rate, washout from the kidney, survival after transplant after either TFF or ultracentrifugation^[^ ^20^ ^]^ is reported here. First, sIONP (1.44 g Fe) was synthesized and separated into two batches (0.72 g Fe each), which were purified by either ultracentrifugation^[^ ^20^ ^]^ or TFF. In the TFF purification process, the sIONP solution was diluted to a concentration of 0.72 mg Fe/mL and then transferred into the feed reservoir through the feed pump. The sIONP were passed tangentially through polysulfone (PS) hollow‐fiber filters under pressure. Simultaneously, ultra‐pure water was continuously introduced into the feed reservoir from the external reservoir via the feed pump. The filtrates were collected into the filtration collection vessel and discarded. This continuous dynamic process resulted in a reduction of the permeate pH level from 13–14 to 6.5–7.0 after running 12 L of ultra‐pure water through 0.72 g of sIONP for 2 h and 30 min, using the TFF parameters listed in Table S2 (Supporting Information). Before initiating the TFF procedure, the system was primed by circulating 5 L of ultra‐pure water to prevent air pockets and enhance the filtration flux rate. In contrast, the sIONP solution (0.72 g Fe) was purified using the seven‐step ultracentrifugation method outlined in reference,^[^ ^20^ ^]^ which required 2 L of ethanol, 630 mL of ultra‐pure water, 12 centrifuge tubes (26×77 mm, 29.9 mL capacity), and 3 days of purification time. Immediately after purification by TFF or ultracentrifugation, the sIONP solutions were concentrated, characterized, and evaluated.

### 2.2 Characterization of Purified sIONP

DLS and *ζ*‐potential measurements demonstrated that the TFF process did not significantly impact the coating of the sIONP. Based on DLS, TFF‐purified sIONP had a size of 88 ± 5 nm, compared to the ultracentrifuged sIONP, which had a size of 93 ± 4 nm, as shown in Figure S1 (Supporting Information); this difference is not statistically significant (*p* = 0.210). Due to the proprietary coating of the EMG 308, the *ζ*‐potential exhibited a negative value of −50 ± 1 mV, which, in the presence of non‐ionic PEG coating, reduced to −37 ± 1 mV for ultracentrifuged sIONP and −41 ± 2 mV for TFF‐purified sIONP (Table S2, Supporting Information), showing no statistically significant difference (*p* = 0.204). Notably, the TFF purification process did not negatively affect the coating on the sIONP, as evidenced by the DLS and *ζ*‐potential values. TEM images in Figure S2 (Supporting Information) indicated that sIONP purified by TFF (*n* = 10) measured 47 ± 3 nm, while ultracentrifuged sIONP measured 49 ± 1 nm, with no statistically significant difference (*p* = 0.244).

### 2.3 Colloidal Stability

**Figure 2:**
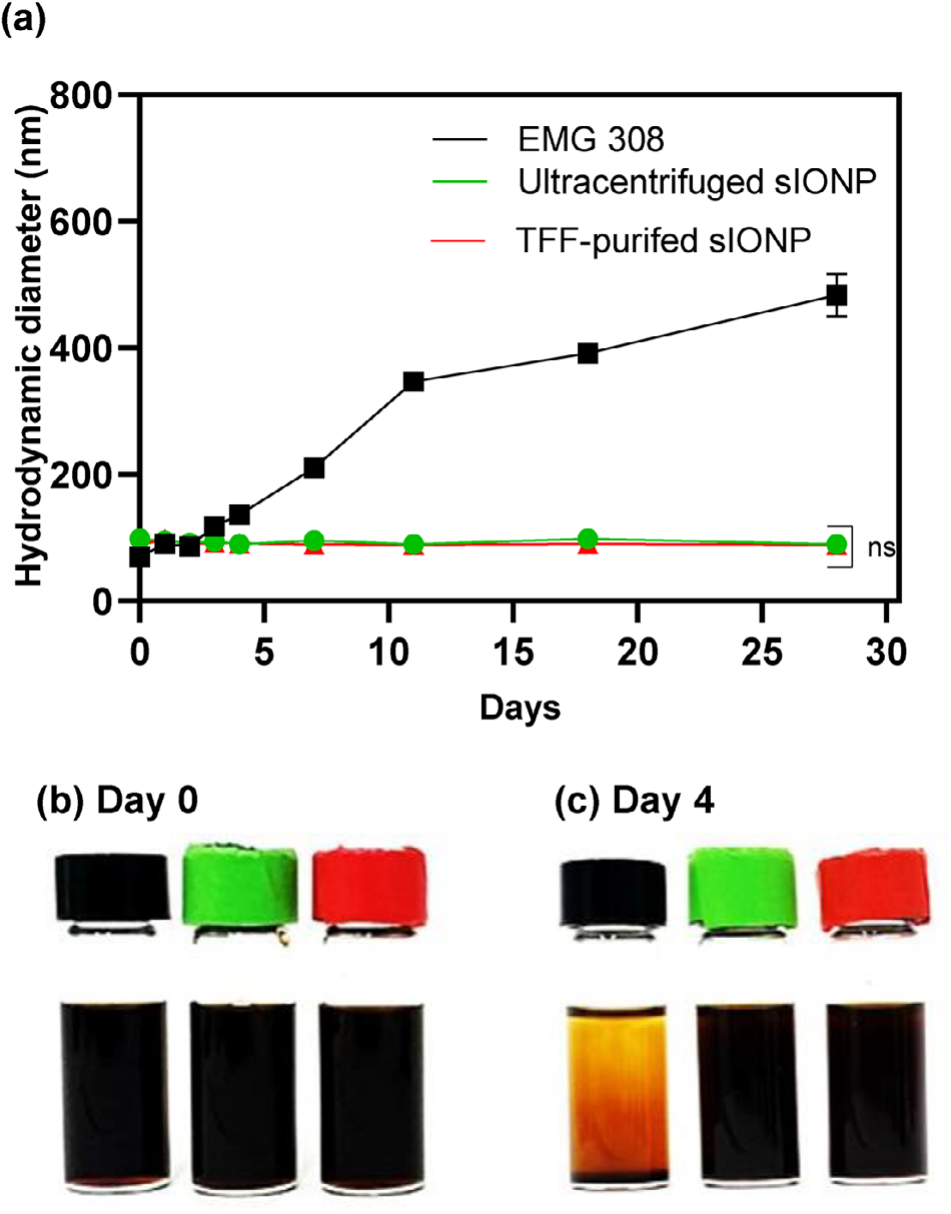
Stability of EMG 308 and sIONP (1 mgFe/mL) in VMP solution. a) DLS sizes of EMG 308, ultracentrifuged sIONP, and TFF‐purified sIONP for 4 days. b,c) Photographs of EMG 308 (black), ultracentrifuged sIONP (green), and TFF‐purified sIONP (red) b) immediately after and c) 4 days after the addition to VMP solution. Data represents mean ± SD. *n* = 3 replicates per group. The two‐tailed *t*‐test was used for statistical comparison between ultracentrifuged and TFF‐purified sIONP on day 28. ns, not significant

The stability of IONPs in CPAs is crucial for cryopreservation and nanowarming applications. Unstable IONPs could result in aggregation in the vasculature of organs, unloading inefficiencies,^[^ ^20^ ^]^ and a drop in heating.^[^ ^24^, ^31^ ^]^ As a part of our study, we demonstrated the impact of purification method by evaluating the stability of EMG 308 (negative control), ultracentrifuged sIONP, and TFF‐purified sIONP in VMP and VS55 CPAs at an iron concentration of 1 mg mL^−1^. Stability was determined by dynamic light scattering measurement over time. Upon the addition of the nanoparticles to the VMP, there was no immediate increase in size or visual color change (i.e., the solution would become lighter if IONP aggregates and settles). Notably, EMG 308 exhibited a size increase to 118 ±  10 nm on the third day, which was followed by precipitation and a color change from dark brown to light brown on the fourth day. In contrast, ultracentrifuged (90 ± 3 nm) and TFF‐purified sIONP (89 ± 3 nm) remained stable without a significant increase in size (*p* = 0.708) or color change even after several days (**Figure** **2**). This implies that TFF purification sufficiently preserves the coating on IONPs to avoid precipitation in CPAs. Next, we demonstrated stability in VS55, a common CPA cocktail.^[^ ^20^ ^]^ In contrast to VMP, EMG 308 is less stable in VS55 and showed an increase from 70 ± 0.21 nm to 432 ± 6 nm after only 45 min, which was followed by a color change from brown to light brown due to precipitation (Figure S3, Supporting Information). On the other hand, there was no color change, precipitation, or statistical difference between the sizes of ultracentrifuged sIONP (80 ± 3 nm) and TFF‐purified sIONP (81 ± 3 nm) up to day 4 (*p* = 0.508). Both ultracentrifuged sIONP and TFF‐purified sIONP remained stable even after 7 months in VMP and VS55.

### 2.4 Heating Characterization and Raman Spectroscopy

**Figure 3:**
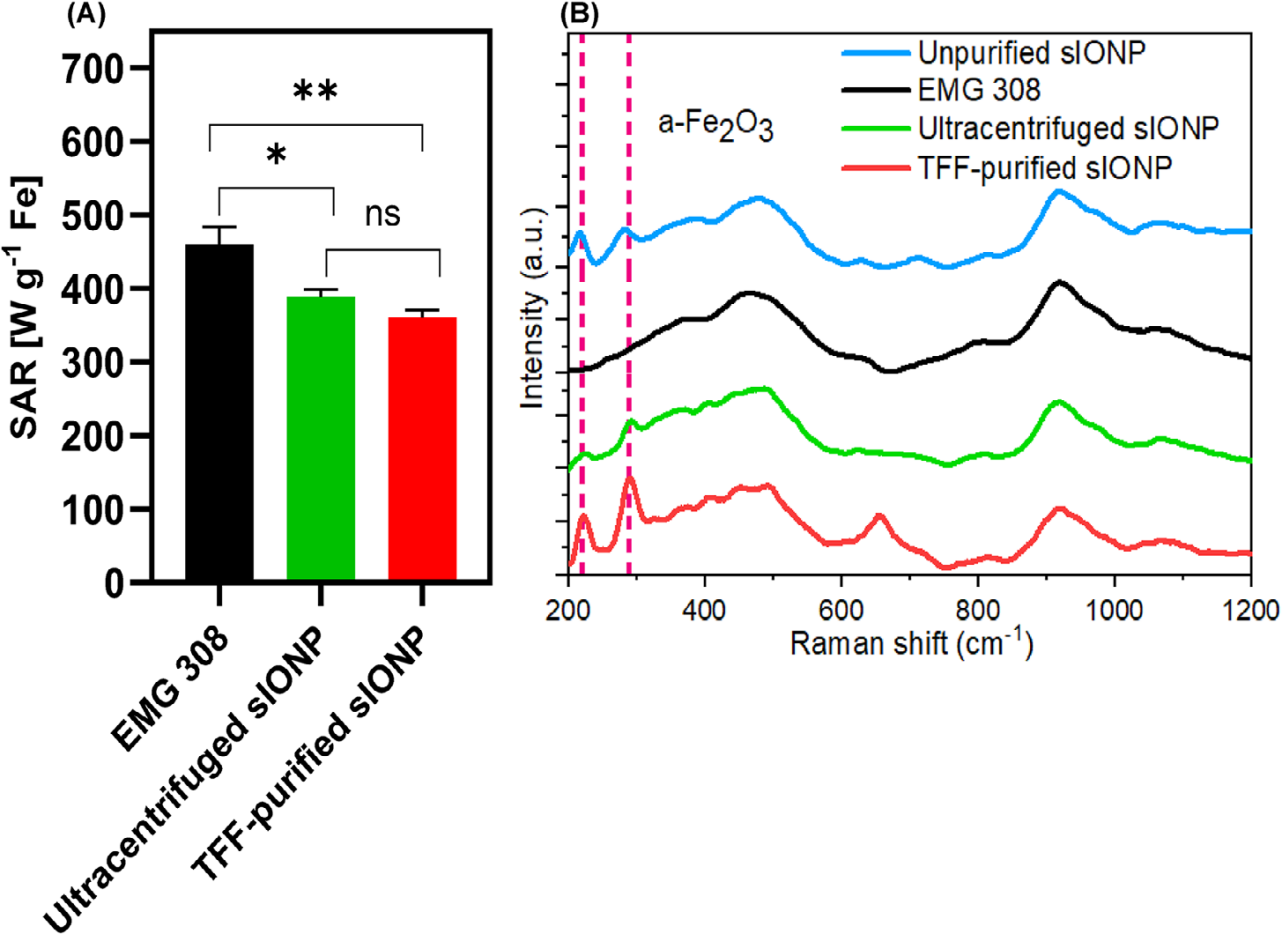
Heating and Raman spectra of different IONP formulations. a) Specific absorption rate of units in weight (SAR_Fe_) at 360 H_Z,_ 20 KAm^−1^ for EMG 308, ultracentrifuged sIONP, and TFF‐purified sIONP in water. Data represents mean ± SD. One‐way ANOVA followed by Tukey's post hoc test was used for statistical comparison. *n* = 6 replicates per group. ns; not significant, ^*^ *p* < 0.05, ^**^ *p* < 0.01, b) Raman spectra for EMG 308, ultracentrifuged sIONP, and TFF‐purified sIONP in water.

IONPs are selected for nanowarming to heat CPA systems and organs volumetrically. Thus, it is imperative to evaluate whether the sIONP purification method impacts heating performance. To assess this, we measure the heat generation or specific absorption rate per g of iron in each formulation (SAR_Fe_) at 360 H_Z,_ 20 KAm^−1^ in water at a concentration of 4 mgFe mL^−1^ (**Figure** **3**a). The results showed that EMG 308 had the highest SAR, while both ultracentrifuged sIONP and TFF‐purified sIONP showed an expected slight decrease in SAR, previously attributed to core–coating interactions.^[^ ^20^ ^]^ Nevertheless, these SAR values were not statistically different between ultracentrifuged and TFF‐purified sIONP (*p* = 0.452). However, a statistically significant difference was observed between EMG 308 and ultracentrifuged sIONP (*p* = 0.019) and between EMG 308 and TFF‐purified sIONP (*p* = 0.0016). Furthermore, Raman spectroscopy was utilized to ascertain if there were any variations in the chemistry of the IONP core for EMG 308, before and after purification. Spectra were collected for the unpurified sIONP, EMG 308, ultracentrifuged sIONP, and TFF‐purified sIONP in water (Figure 3b). Characteristic vibrational modes expected to be Raman‐active for magnetite (Fe_3_O_4_) were observed at peaks around ≈490 and ≈630 cm^−1^^[^ ^32^, ^33^ ^]^ for EMG 308, unpurified sIONP, ultracentrifuged sIONP, and TFF‐purified sIONP, indicating that the magnetite core structure remained intact throughout the purification process (Figure 3b).

However, unpurified, ultracentrifuged, and TFF‐purified sIONP showed weak peaks around ≈230 and ≈290 cm^−1^, which strongly indicate the presence of vibrational modes from the Raman‐active hematite (α‐Fe_2_O_3_).^[^ ^34^, ^35^ ^]^ Although hematite is stable, it is weakly magnetic with antiferromagnetic behavior, leading to a reduction in the saturation remanent magnetization of the IONP. The reduction in SAR values observed in both TFF and ultracentrifuge purified samples, along with Raman spectroscopy indicating the emergence of α‐Fe_2_O_3_, suggests that oxidation of the IONP core likely occurred during synthesis and purification. This finding aligns with previous reports that magnetite (Fe_3_O_4_) exhibits higher saturation magnetization than its oxidized forms, such as maghemite (γ‐Fe_2_O_3_).^[^ ^36^ ^]^ Oxidation of the core can thus contribute to the observed decrease in saturation remanent magnetization (and SAR) of sIONP. Future optimization of the coating process may help reduce or prevent this oxidation and the resulting decrease in the saturation remanent magnetization (and SAR) of sIONP, thus preserving the magnetic properties of the sIONP, thereby improving their heating efficiency.

### 2.5 HRMAS NMR Analysis

One benefit of the silica coating is the ability to attach PEG, which adds stability in biological fluids and CPAs.^[^ ^20^, ^25^ ^]^ To assess the presence of the PEG layer on the surface of sIONP after purification, HRMAS NMR was performed. Samples were prepared using our reported method,^[^ ^19^ ^]^ and peaks at 3.66–3.76 ppm (corresponding to methylene protons close to the chain ends of PEG) were evaluated and shown in Figure S4 (Supporting Information). PEG peak signals were present from both ultracentrifuged sIONP and TFF‐purified sIONP, suggesting an intact PEG layer even after purification (Figure S4, Supporting Information). Notably, the stability of TFF‐purified sIONP was statistically similar to ultracentrifuged sIONP in CPAs (Figure 2; Figure S3, Supporting Information).

### 2.6 sIONP toxicity on HDF cells

**Figure 4:**
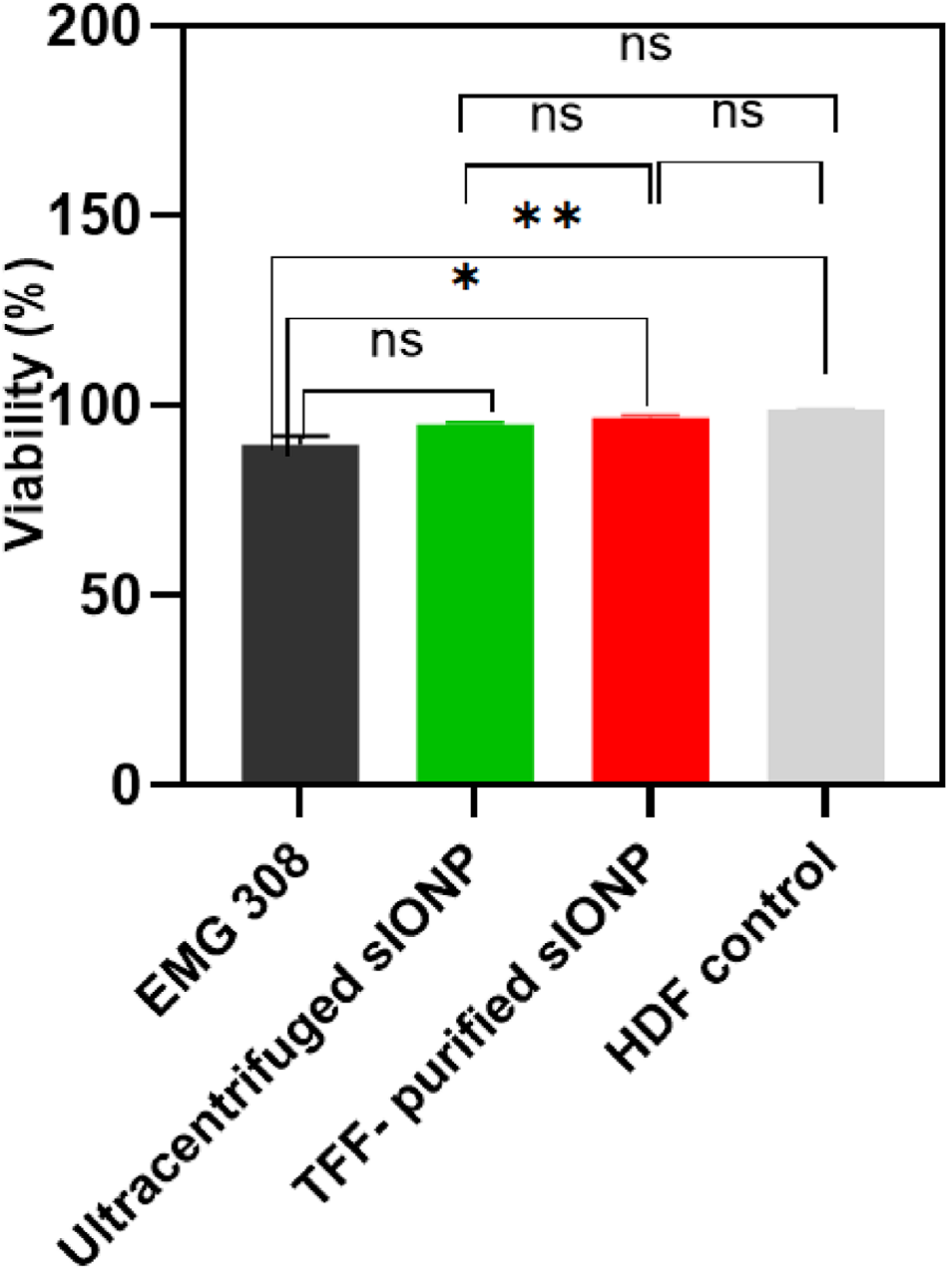
Biocompatibility of different IONP formulations. Viability assessment of EMG 308, ultracentrifuged sIONP, and TFF‐purified sIONP after 24 h of exposure to HDF cells at 10 mg Fe mL^−1^. Data represent mean ± SD. One‐way ANOVA followed by Tukey's post hoc test was used for statistical comparison. *n* = 3 biological replicates per group. ns; not significant, ^*^ *p* < 0.05, ^**^ *p* < 0.01.

The cytotoxicity of TFF‐purified sIONP was evaluated using HDF cells at a concentration of 10 mgFe mL^−1^ and compared to previous data in HDF using ultracentrifuged sIONP.^[^ ^20^ ^]^ **Figure** **4** results showed TFF‐purified sIONP had a high cell viability of 96.7% ± 1%, which was not statistically different from ultracentrifuged sIONP, with 95.0% ± 1% viability (*p* = 0.754), or from the HDF control, 98.97% ± 0.06% viability (*p* = 0.544). However, EMG 308 showed the lowest viability of 89.67% ± 3.79%. and statistically different from HDF control (*p* = 0.0023) and TFF‐purified sIONP (*p* = 0.013). Statistical similarities were shown between EMG 308 and ultracentrifuged sIONP (*p* = 0.050), and ultracentrifuged sIONP and HDF control (*p* = 0.159). This suggests intact non‐ionic biocompatible PEG on sIONP after purification conferred no toxicity to the sIONP at 10 mgFe mL^−1^ for 24 h after exposure time.

### 2.7 Perfusion CPA Loading and Vitrification of Kidney

**Figure 5:**
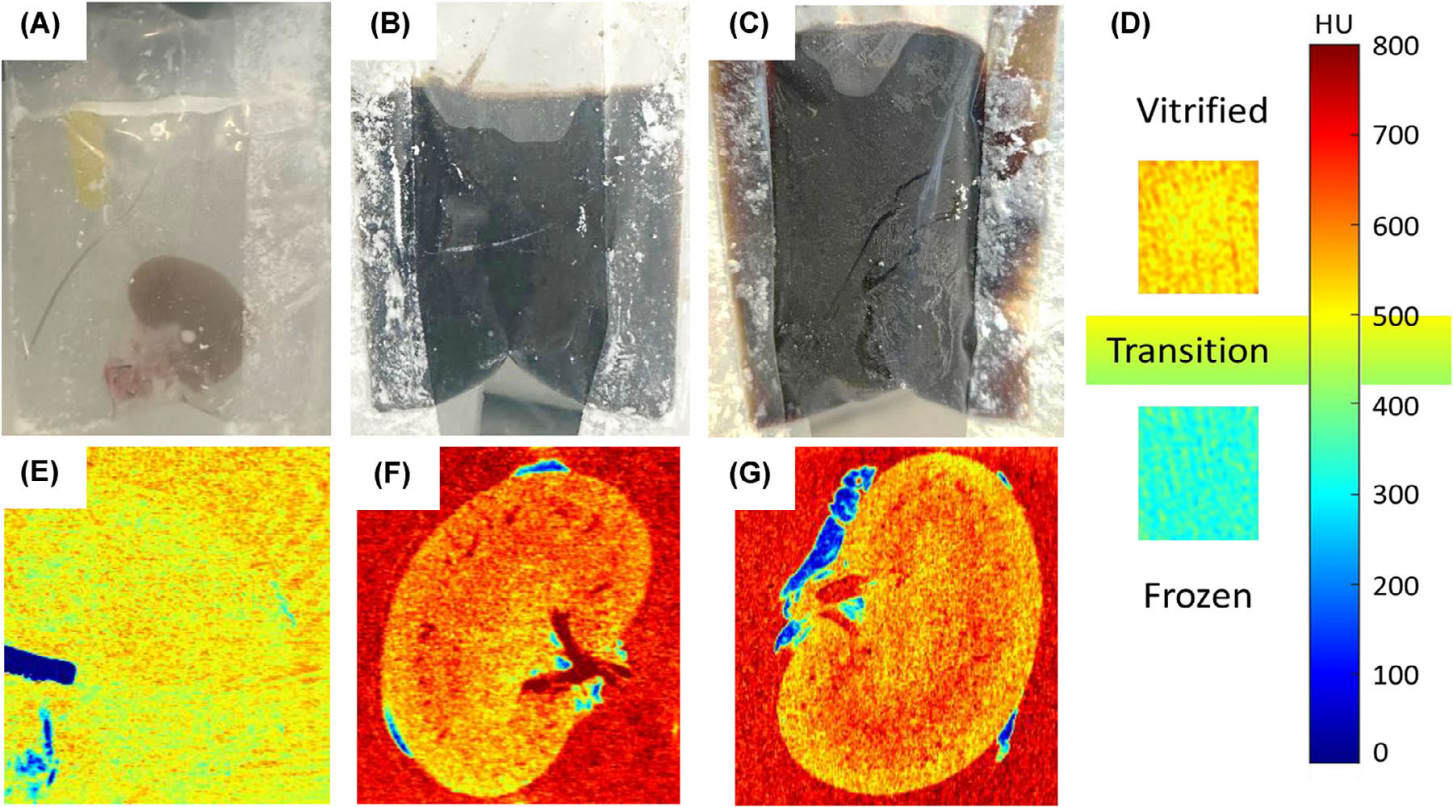
Photographs and Micro‐computed tomography of vitrified kidneys with and without IONP loading. Top row: photographs of polyethylene bags containing vitrified kidneys loaded with a) VMP (CPA control), b) Ultracentrifuged sIONP in VMP, and c) TFF‐purified sIONP in VMP, d) Pseudo color scale represents micro‐CT image radiodensity in Hounsfield units (HU). Bottom row: e,f,g) micro‐CT pseudo color images of vitrified kidneys in the top row (a), (b), and (c), respectively. The HU scale differentiates between frozen and vitrified kidneys based on attenuation. Low attenuation of ˂ 400 HU is indicative of a frozen kidney, while > 500 HU indicates a vitrified kidney^[^ ^3^, ^37^ ^]^

Our previous VMP protocol^[^ ^3^ ^]^ was used for perfusion loading the CPA along with ultracentrifuged and TFF‐purified sIONP into the kidneys. Figure S5 (Supporting Information) shows the arterial pressure and flow rate during perfusion. sIONP were loaded at a concentration of 10 mgFe mL^−1^ immediately after CPA perfusion, and each kidney was inserted into a polyethylene bag filled with 15 mL full‐strength VMP solution (8.4 m) containing 4 mg Fe mL^−1^. TFF‐purified sIONP showed a loading pressure between 30 and 40 mmHg, while ultracentrifuged sIONP were between 40 and 50 mmHg at a constant flow rate of 0.5 mL min^−1^. Figure S6 (Supporting Information) shows the thermal history recorded by a fiber‐optic temperature probe placed near the kidney. Organ cooling and vitrification followed our previous protocol,^[^ ^3^ ^]^ and the organ was quickly moved to the −150 °C storage freezer. Figure S6 (Supporting Information) shows at the cooling rate for the TFF‐purified sIONP (21.12 ± 6.02 °C min^−1^) was not significantly different from ultracentrifuged (20.5 ± 8.1 °C min^−1^)^[^ ^3^ ^]^ (*p* = 0.896) and faster than the CCR of VMP in kidney tissue (2 °C min^−1^).^[^ ^3^ ^]^ Micro‐computed tomography (micro‐CT) and visual inspection of the vitrified kidney sample in the polyethylene bag were performed to assess vitrification. **Figure** **5**a shows the translucent, glassy appearance of the VMP solution surrounding the CPA‐vitrified kidney. The deep brown color of the sIONP solution in the kidneys loaded with ultracentrifuged and TFF‐purified sIONP prevented complete visualization of the kidneys (Figure 5b,c). However, the translucent, glassy appearance of the surrounding solution and the kidney's lack of a pale pink color indicate successful vitrification of the TFF‐purified sIONP. Furthermore, micro‐CT was utilized based on radiodensity (represented in Hounsfield units (HU)) to differentiate frozen and vitrified tissues.^[^ ^3^, ^37^ ^]^ The HU units are read based on attenuation. A low attenuation of ˂ 400 HU indicates a frozen kidney, while > 500 HU indicates a vitrified kidney. The CPA‐loaded kidney (no IONP) had a radiodensity of 500 ± 41 Hounsfield units (HU) (Figure 5e), similar to the previously published vitrified kidneys.^[^ ^3^ ^]^ Following loading with IONP, the HU values increased for both ultracentrifuged and TFF‐purified sIONP‐loaded kidneys (Figure 5f), aligning with our earlier findings (Figure S7, Supporting Information) across all kidney regions. No areas of low attenuation, indicating cracking or ice crystallization, were detected in any of the kidneys, confirming successful vitrification. The low attenuation/blue color observed outside the kidney was limited to perihilar fatty tissues, likely due to lower CPA concentration in these less well vascularized regions, and is of minimal significance. Notably, HU values suggest that TFF‐purified sIONP achieved vitrification within VMP CPA and kidneys similarly to ultracentrifuged sIONP (Figure 5e; Figure S7, Supporting Information).

### 2.8 Nanowarming and Unloading of CPA/IONPs From the Kidney

A 15 kW radiofrequency (RF) coil operating at 94% power, with a field strength of 63 kA m^−1^ and a frequency of 180 kHz, was used for nanowarming of the vitrified kidney.^[^ ^3^ ^]^ The warming rate adjacent to the kidneys for the TFF sIONP–loaded kidney (*n* = 4) was 76.55 ± 1 °C min^−1^, which was not statistically significantly different when compared to rewarming rates from previously published ultracentrifuged sIONP‐loaded kidney (*n* = 6, 72.0 ± 8.0 °C min^−1^)^[^ ^3^ ^]^ at *p* ˂ 0.05 (*p* = 0.161). This rate was also above the CWR of VMP (≈50 °C min^−1^) in kidney tissues, and capable of preventing devitrification and cracking (Figure S8a, Supporting Information). Immediately after nanowarming, the CPA and IONPs were perfusion unloaded following our previous perfusion protocols. Arterial pressure and flow during the unloading of kidneys with centrifuged and TFF‐purified sIONP are shown in Figures S8b,c (Supporting Information). Post‐assessment evaluation of the kidneys was performed by histologic and transplantation studies.

### 2.9 Histology

**Figure 6:**
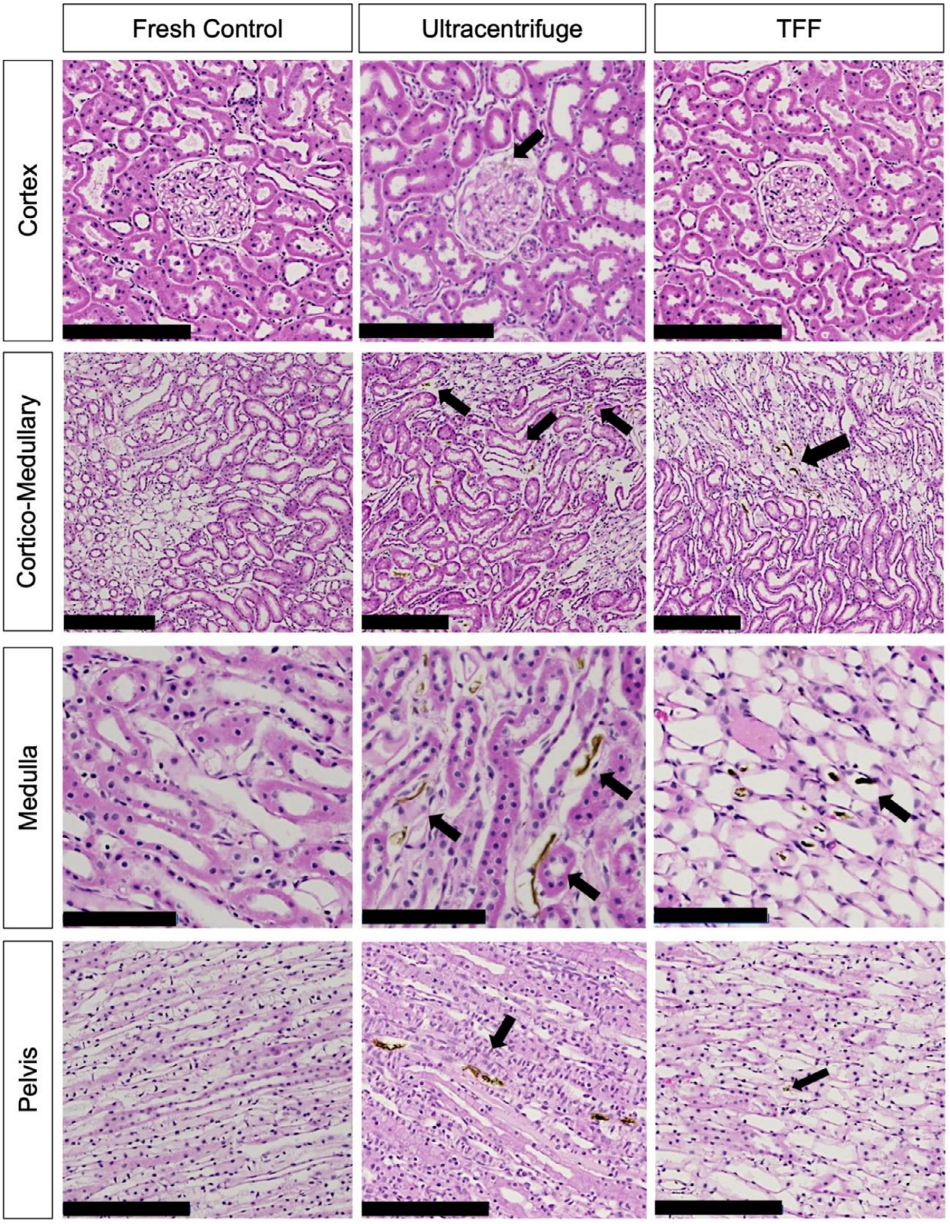
Histologic appearance of fresh control and cryopreserved kidneys. Representative histology of fresh control kidneys, TFF, and ultracentrifuged vitrified nanowarmed kidneys after loading and unloading CPA‐nanoparticle cocktail across cortex, cortico‐medullary junction, medulla, and pelvis. Scale bars are set at 200, 250, 100, and 200 µm for cortex, cortico‐medullary junction, medulla, and pelvis, respectively. The black arrows indicate the brown residual, potentially linked to IONPs, in different regions of the kidney.

Fresh control kidneys ultracentrifuged and TFF‐ purified sIONP‐ loaded, vitrified, nanowarmed, and unloaded kidneys were bisected and assessed for histological changes. Visual inspection of the bisected kidneys revealed near‐normal gross architecture with no macroscopic residues or ice damage in either ultracentrifuged or TFF‐ purified sIONP vitrified and nanowarmed cases, both comparing favorably to fresh control kidneys stored in UW solution. Staining 4 µm sections of the kidney with H&E microscopy demonstrated small amounts of brown residue in regions of the medulla and pelvis, particularly in the ultracentrifuge sIONP kidneys, consistent with observations in prior work (compared in **Figure** **6** ^[^ ^3^ ^]^). This brown residue was either localized in the capillaries or had extravasated into the interstitial space. The largest residues were found in the medulla and cortico‐medullary junction (Figures 6, 8; Figure S10, Supporting Information). However, no residue was greater than 50 µm in the cortex or pelvis of kidneys perfused with either ultracentrifuged or TFF‐purified sIONP (Figures 6, 8; Figure S10, Supporting Information). Residues ranging from 10 to 50 µm were found in the glomeruli, Bowman's space, and proximal and distal convoluted tubules in both TFF and ultracentrifuge groups. The corticomedullary junction and inner medulla had the most residues (Figures 6, 8; Figure S10, Supporting Information). However, the ultracentrifuge group exhibited significantly higher residue content compared to the TFF group in multiple kidney regions: the cortex (10–50 µm, *p* = 0.04), cortico‐medullary junction (≤10 µm, *p* = 0.0003; >50 µm, *p* = 0.024), and pelvis (10–50 µm, *p* = 0.024) (Figure 8).

Fresh control kidneys did not demonstrate any brown residues. The glomeruli showed no pathological findings and maintained intact basal membrane, Bowman's space, and endothelial lining in both the TFF, and ultracentrifuge groups compared to fresh controls. Proximal and distal convoluted tubules exhibited near‐normal histological findings without evidence of tubular necrosis in either group. The tubules in the medulla and collecting ducts showed no signs of histological changes due to ice injury (Figure 6).

Based on well‐established principles of tangential flow filtration (TFF), we attribute the reduced residue content observed with TFF to its size‐selective diafiltration mechanism. Unlike ultracentrifugation method, the crossflow dynamics in TFF minimize fouling and aggregation, enabling consistent and scalable purification. In our study, the use of a 100 kDa molecular weight cut‐off membrane under optimized flow and pressure conditions allowed the effective removal of free polymers, silica byproducts, and unreacted components, while retaining the IONP. This mechanism likely accounts for the enhanced residue reduction observed with TFF compared to ultracentrifugation.

### 2.10 Iron Quantification in the sIONP Unloaded Kidneys

Efficient unloading of sIONP after perfusion is crucial for clinical applications and the translatability of nanowarming. Figure S9 (Supporting Information) shows the residual Fe concentration in the kidney after unloading CPA and IONPs from control, ultracentrifuged, and TFF‐purified sIONP. There was no statistically significant difference between the ultracentrifuged and TFF kidneys (*p* = 0.819), control and ultracentrifuged kidneys (*p* = 0.192), or control and TFF kidneys (*p* = 0.354). The total amount of iron was 0.26 ± 0.07 µgFe mg^−1^ dry tissue (control), 1.62 ± 1.27 µgFe mg^−1^ dry tissue (ultracentrifuged kidneys), and 1.23 ± 0.78 µgFe mg^−1^ dry tissue (TFF kidneys). This amount is similar to previous work which led to no adverse effects (2.0 µgFe mg^−1^ dry tissue).^[^ ^20^ ^]^ iron drug solutions. Of note, numerous iron based drugs and imaging agents are routinely given intravenously up to 2.6 mg/kg ^[^ ^38^ ^]^

### 2.11 Transplantation

**Figure 7:**
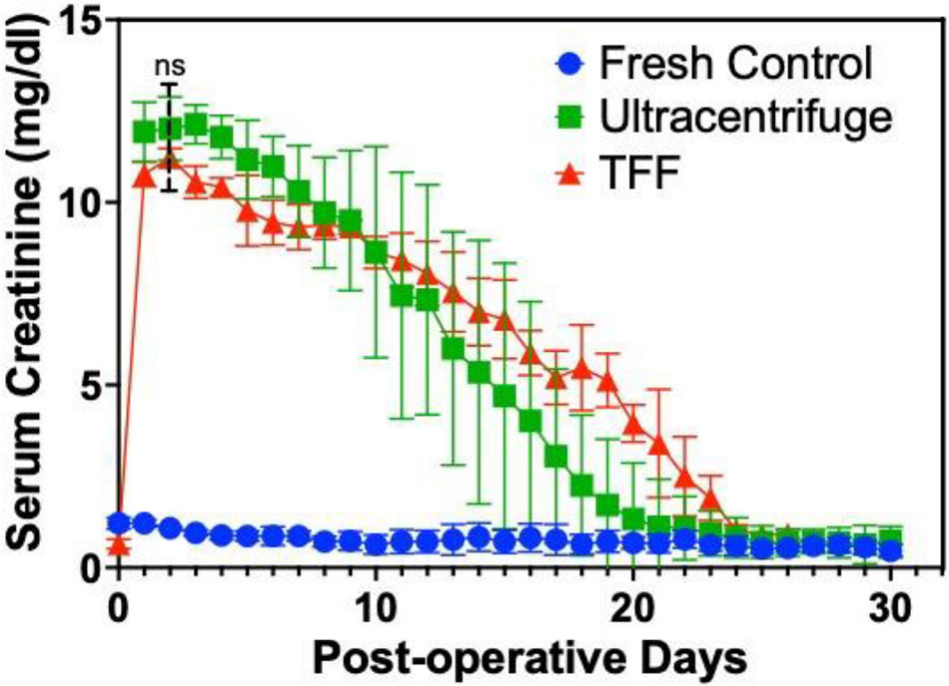
Serum creatinine following transplantation of vitrified rewarmed kidneys. TFF (*n* = 4; red) and ultracentrifuge (*n* = 6; green) vitrified‐nanowarmed kidney transplants and fresh control transplants (*n* = 6; blue) from our previous study.^[^ ^3^ ^]^ Data represents mean ± SD. One‐way ANOVA followed by Tukey's post hoc test was used for statistical comparison. ns; not significant in peak serum creatinine level between ultracentrifuged sIONP and TFF‐purified sIONP on day 2.

**Figure 8:**
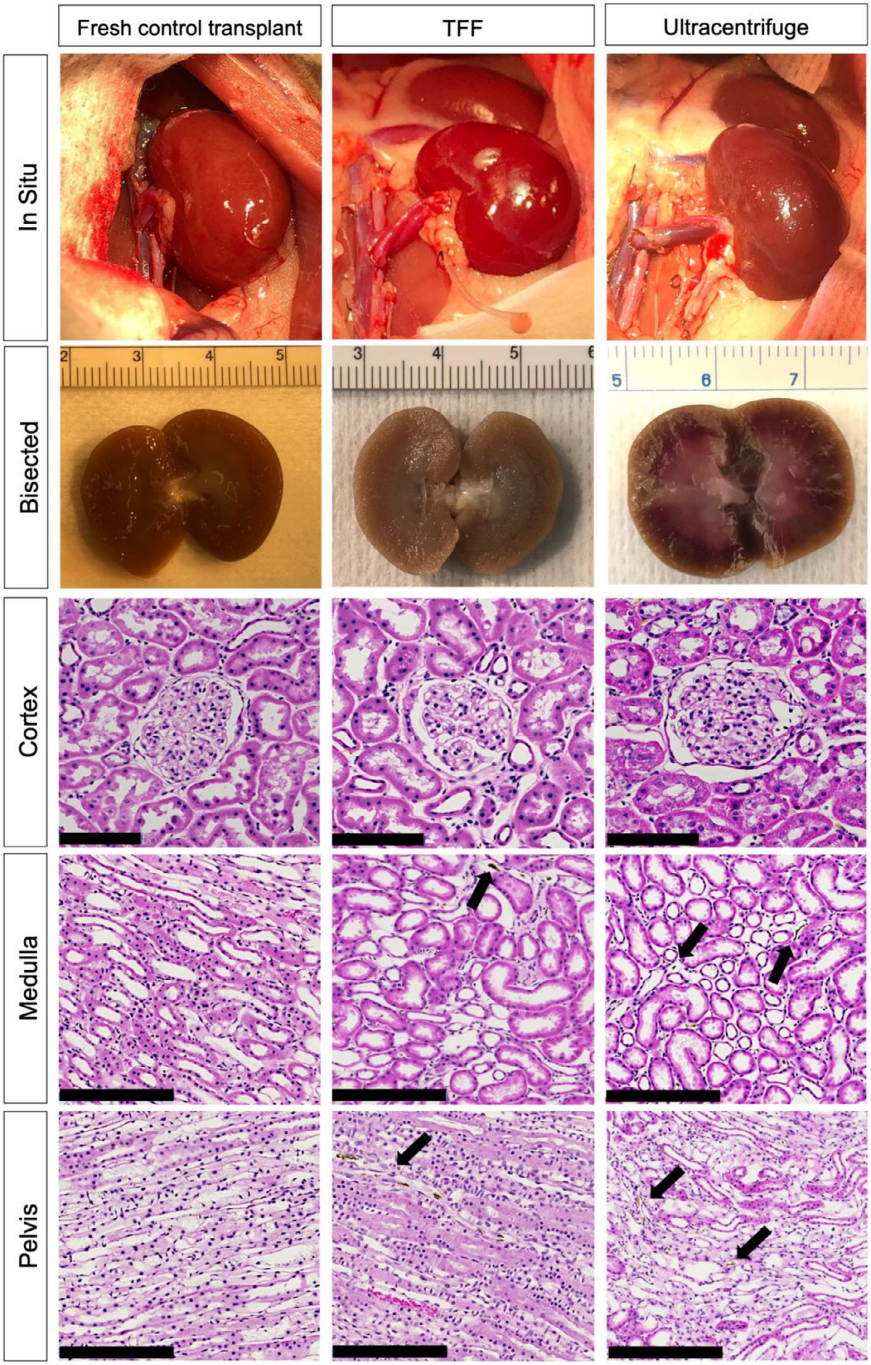
Gross and histologic appearance of vitrified nanowarmed kidneys. Reperfusion appearance in situ of fresh control, TFF, and ultracentrifuge vitrified nanowarmed kidneys at the time of transplant; Bisected kidneys at explant – Post‐Operative Day (POD) 26 for TFF and POD 30 for Ultracentrifuge from our previous study;^[^ ^3^ ^]^ H&E of renal cortex, medulla, and pelvis with arrows pointing to residues in the kidneys. Scale bars are 100, 200, and 200 µm for the cortex, medulla, and pelvis, respectively.

The outcomes of vitrified and nanowarmed kidney transplants in rats were compared to assess the impact of TFF vs. ultracentrifuged IONP on the outcome. Four syngeneic kidney transplants with TFF particles were compared to six ultracentrifuged and six fresh control kidney transplants from our previous study.^[^ ^3^ ^]^ Kidneys from all groups were reperfused immediately and homogeneously upon releasing the clamps and restoration of blood flow (Figure 8). Fresh control kidneys produced urine immediately at reperfusion, while all nanowarmed kidneys produced urine within 30 min. No vascular thrombosis was detected in any of the transplants. Serum creatinine levels from TFF vitrified‐nanowarmed kidney transplants were monitored until normalization for a minimum of three consecutive days (Day 26). Serum creatinine levels in all nanowarmed kidney recipients peaked between 2 and 5 postoperative days (POD). The peak serum creatinine in the TFF and ultracentrifuge vitrified nanowarmed groups were 11.23 ± 0.22 and 12.17 ± 0.52 mg dL^−1^ respectively which were not statistically different (*p* = 0.122). Creatinine levels in the fresh control transplant group were significantly lower than the vitrified nanowarmed groups (*p* < 0.0001). Creatinine levels in the vitrified‐nanowarmed groups gradually declined after peaking to reach normal levels over 2–3 weeks (**Figure** **7**) with no statistical difference at POD 26. These results were consistent with prior studies.^[^ ^3^ ^]^ Note, we excluded three kidneys: one due to a technical failure related to anesthesia during surgery, another due to issues with perfusion, and the third due to the recipient's age.

**Figure 9:**
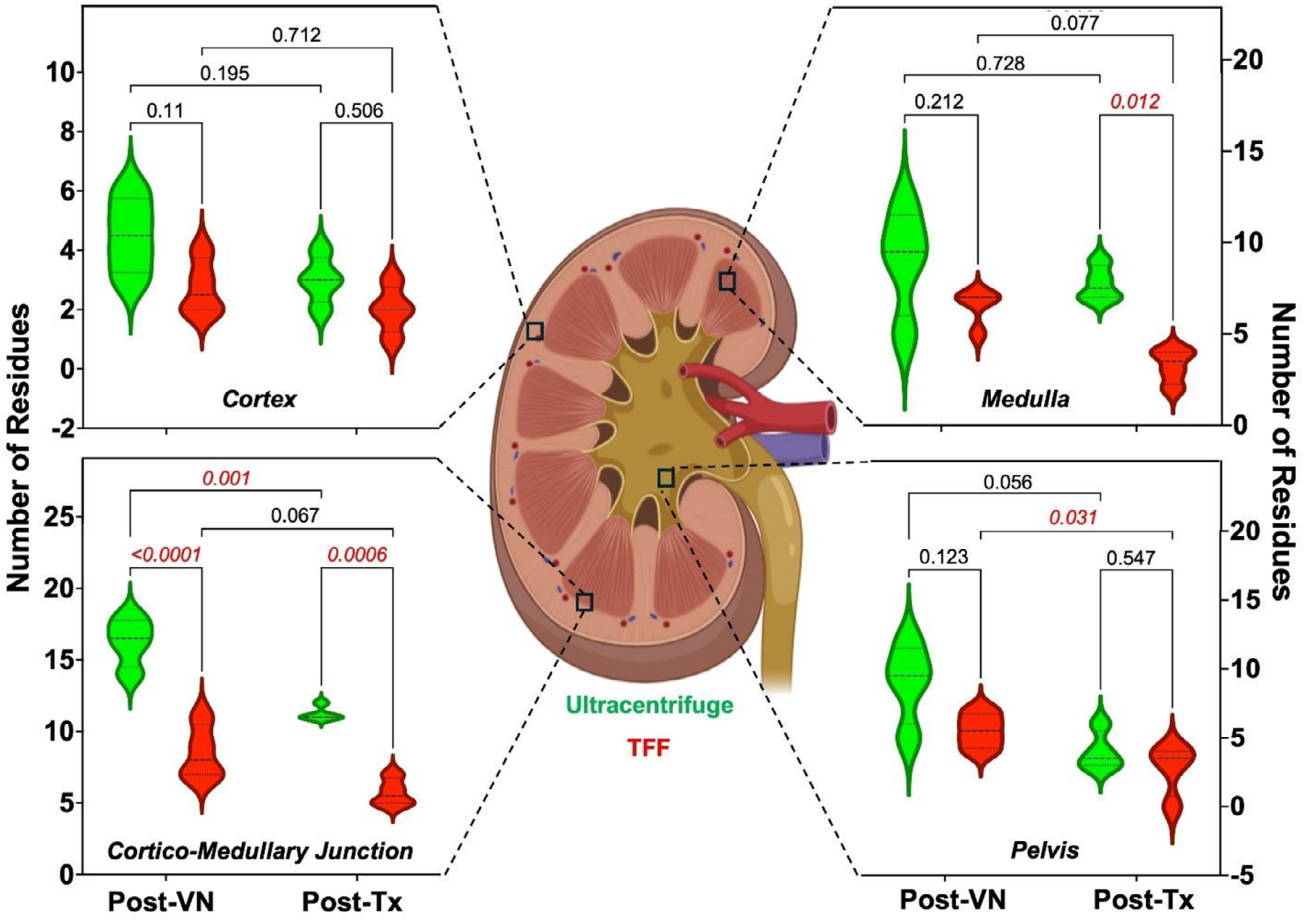
Residue distribution per region on microscopy at explant following transplantation. Number of brown residues (N) per field of view (*n* = 4) in the cortex, cortico‐medullary junction, medulla, and renal pelvis in the TFF (*n* = 4; red) and ultracentrifuge groups (*n* = 6; green) after 26 days in the TFF group and 30 days in ultracentrifuge group^[^ ^3^ ^]^ Data represents mean ± SD. One‐way ANOVA followed by Tukey's post hoc test was used for statistical comparism. ^*^ *p* < 0.05, ^**^ *p* < 0.01, ^***^ *p* < 0.001

TFF vitrified nanowarmed kidneys were explanted on POD 26 and demonstrated normal gross anatomy and appearance when bisected, similar to that of fresh control transplants and ultracentrifuge vitrified nanowarmed kidneys (**Figure** **8**).^[^ ^3^ ^]^ Irrespective of the type of sIONP purification method, histological assessment of transplanted vitrified‐nanowarmed kidneys showed minimal focal tubular necrosis with intact basement membranes and vasculature. There were no microthrombi or distal infarcts in any of the groups. Both vitrified‐nanowarmed and fresh control kidneys exhibited normal morphology of large blood vessels and collecting ducts. (Figure 8). Residue accumulation in the medulla and pelvis of nanowarmed kidneys was assessed using H&E staining. Significantly higher residue levels were observed in the cortico‐medullary junction of ultracentrifuged kidneys, both post‐vitrification nanowarming (p‐VN) (*p* < 0.0001) and post‐transplant (p‐TX) (*p* = 0.0006), as well as in the medullary region post‐transplant (*p* = 0.012), compared to TFF kidneys (**Figure** **9**b). No significant differences were observed in the cortex (post‐VN: *p* = 0.11; post‐TX: *p* = 0.506) or pelvis (post‐VN: *p* = 0.123; post‐TX: *p* = 0.547). (Figure 9). Furthermore, we performed Prussian blue staining of kidneys that have been vitrified and nanowarmed using an ultracentrifuge and TFF‐purified sIONP. As shown in Figure S11 (Supporting Information), Prussian blue staining was positive in the kidney renal cortex in some sections, as seen within the glomerular tuft and in the interstitial space, before and after transplantation for either ultracentrifuge or TFF‐purified sIONP kidneys. While our previous work correlated brown residues with sIONP deposition following sIONP‐VS55 load/ unload only,^[^ ^18^ ^]^ in this study we demonstrate Prussian blue staining of biologically compatible VMP‐sIONP cocktail for both ultracentrifuge and TFF‐purified sIONP before and after transplantation (Figure S11).

## 3 Conclusion

In summary, we have demonstrated the tangential flow filtration (TFF) method for purifying silica‐coated iron oxide nanoparticles (sIONP) for use in nanowarming. Scaling nanowarming to clinical use will indeed require gram‐level quantities of IONPs per organ. For example, a human kidney, with an estimated vascular fraction of 25% and a volume of ≈200 mL,^[^ ^39^ ^]^ would require around 500 mg of iron (Fe) when perfused with IONPs at 10 mgFe mL^−1^. This also does not factor in the additional volumes that would be required for perfusion. If all the 25 000 kidneys used for transplants annually in the U.S. were first nanowarmed, the total IONP demand would amount to approximately 12.5 kg per year. This requirement increases substantially when factoring in additional transplantable organs such as the liver and heart, where nanowarming has also been attempted. Conventional purification approaches, such as ultracentrifugation become impractical at this scale due to throughput and operational limitations. In contrast, the tangential flow filtration (TFF) method demonstrated in this work is inherently scalable and compatible with a wide range of production volumes. While our current lab‐scale purification was performed on 0.72 g Fe, TFF systems are routinely used in bioprocessing to handle volumes ranging from milliliters to several thousand liters. For instance, commercial platforms such as the Sartoflow 4500 can routinely process 200–2000 L of unfiltered solutions yielding between 1 and 15 kg of filtered products (e.g. IONP).

TFF was evaluated in comparison to conventional ultracentrifugation, which was previously reported for sIONP. The results showed no statistically significant differences between ultracentrifuged sIONP and TFF‐purified sIONP in terms of stability in VMP or VS55, core heating capabilities (evident from SAR values), viability of HDF cells after IONP exposure, DLS size, or zeta potential of the sIONP. Furthermore, the perfusion of sIONP into rat kidneys demonstrated sufficient distribution for vitrification and nanowarming with ultracentrifuged sIONP and TFF‐purified sIONP. Additionally, transplantation studies indicated comparable survival and kidney function, with serum creatinine levels converging between ultracentrifuged sIONP and TFF‐purified sIONP with the control group at 26 postoperative days. Interestingly, brown residues were observed in all IONP kidneys after washout (consistent with prior studies) but significantly reduced in the TFF‐purified vs. ultracentrifuge sIONP group. Notably, both TFF and ultracentrifuges IONP kidney residuals reduced further after 26 (TFF‐purified sIONP) and 30 (ultracentrifuged sIONP) days of transplant, suggesting natural elimination within the body over time. Importantly, in both IONP cases, the remaining Fe content was measured below FDA allowable levels. However, observing this remaining iron and potentially connected brown residues in histology suggests an opportunity for further IONP optimization and further characterization of *in vivo* response to these IONP. Significant advantages of TFF over ultracentrifugation were shown to be the reduction in purification time (2 h and 30 min vs. 3 days), simple path to scalability (i.e., no dependence on vial volumes), elimination of IONP loss, elimination of ethanol use, and reduction in labor. Further investigations will address the slight reduction in SAR values observed between ultracentrifuged and TFF‐purified sIONP, focusing on coating efficiency. Lastly, TFF has shown potential for scaling nanowarming to the clinical organ scale in the near future.

## 4 Experimental Section

### Materials

Ferrofluid EMG308 (Ferrotec, Inc, Bedford USA), 2‐[methoxy(polyethyleneoxy)‐propyl]9‐12‐trimethoxysilane (Gelest, Inc, USA), polyvinylpyrrolidone with a molecular weight of 10 kDa (PVP‐10, Thermoscientific, USA), ethanol (99% and 95%, Decon, USA), tetraethyl orthosilicate (TEOS, Sigma‐Aldrich, USA), chlorotrimethylsilane (TMS, > 99%, Sigma‐Aldrich, USA), ammonium hydroxide (NH_4_OH, 28%, VWR Inc, USA), 2‐(4‐(2‐hydroxyethyl)‐1‐piperazinyl)‐ethanesulfonic acid (HEPES, > 99%, Sigma‐Aldrich, USA), ethylene glycol (> 99%, Sigma‐Aldrich, USA), formamide (> 99%, Sigma‐Aldrich, USA), D (+) Glucose (Fisher, USA), alpha lactose monohydrate (> 99%, Sigma‐Aldrich, USA), potassium chloride (Fisher, USA), sodium bicarbonate (Fisher, USA), dipotassium phosphate trihydrate (> 98%, Spectrum, USA), L‐glutathione (red) (> 98%, Sigma‐Aldrich, USA), adenine hydrochloride (99.9% Chem Impex, USA), X‐1000 (21st Century Medicine, USA), Z‐1000 (21st Century Medicine, USA), dimethyl sulfoxide (> 99%, Sigma‐Aldrich, USA), D‐Mannitol (99.9% Chem Impex, USA), propylene glycol (> 99%, Sigma‐Aldrich, USA), potassium phosphate (> 99%, Sigma‐Aldrich, USA) were all used as received without any further purification.

### Animal Procurement

All animal studies and procedures were conducted in accordance with relevant ethical guidelines and were approved by the Institutional Animal Care and Use Committee (IACUC) at the University of Minnesota (Protocol #2204‐39970 A). Male Lewis rats (Strain #004), aged 16–32 weeks and weighing 450–525 g, were obtained from Charles River Laboratories. Male animals were selected for their larger body size and to avoid potential immunogenicity associated with Y chromosome–encoded antigens. Animals were housed under standard conditions in a conventional facility with a 12 h light/dark cycle, ambient temperature maintained at 68–74 °F, relative humidity between 30% and 50%, and ad libitum access to food (Envigo Lab Diet #2918) and water.

### Synthesis of sIONP

A previously reported method was used to synthesize sIONP.^[^ ^20^, ^40^ ^]^ PVP‐10 (48g) was added to 407 mL of ultra‐pure water at 23 ± 2 °C to dissolve completely. A solution of EMG 308 containing 1.440 g of iron was gently added to the dissolved solution of PVP, making a total volume of 431 mL. The mixture was probe sonicated (Q500, Qsonica) for 4 s on and 2 s off, at 35% amplitude for 45 min under continuous stirring. Thereafter, the mixture was gradually introduced into a = 3.2 L 95% ethanol, and subjected to probe sonication for 45 min, 4 s on and 2 s off, at 35% amplitude with stirring. The mixture was transferred to a 4 L reaction vessel (LG‐8082‐104, Wlmad‐Lab Glass) and stirred by an overhead mechanical stirrer (OS 20‐S Waverly). Ammonium hydroxide (160 mL) was added, followed by 80 mL of TEOS. After 1 h of reaction, PEG‐silane (15 mL) was gently added to the mixture and allowed to react for 30 min. Subsequently, TMS (2.25 mL) was introduced, and the reaction was continued for 24 h under continuous stirring, after which the reaction solution was removed under reduced pressure, and sIONP was collected for immediate purification.

### sIONP Purification by Ultracentrifugation

Synthesized sIONP solution (0.72 g of Fe) was dispensed into 29.9 mL ultracentrifuge tubes and ultracentrifuged at 30 000 rpm for 40 min at 4 °C. The supernatant was gently removed, and the precipitate resuspended in 95% ethanol and left to stand overnight. Thereafter, the centrifuge tubes were bath sonicated until the sIONP fully dispersed in 29 mL ethanol, followed by ultracentrifugation at 30 000 rpm for 20 min at 4 °C. This step was repeated thrice, and the sIONP solution was allowed to sit overnight in 14 mL of ethanol. The following morning, the sIONP were bath sonicated until completely redispersed, and 14 mL of water was added to the centrifuge tubes. The tubes were ultracentrifuged at 30 000 rpm for 25 min at 4 °C, after which the supernatant was carefully discarded. The sIONP were then redispersed in 20 mL of water, after which an additional 9 mL of water was added to the centrifuge tubes and ultracentrifuged again at 30 000 rpm for 40 min at 4 °C on the third day after synthesis. The resulting sIONP were redispersed, filtered, and characterized on day 3.

### sIONP Purification by Tangential Flow Filtration (TFF)

ÄKTA flux TFF system (GE Healthcare Bioscience Corp, USA) and Xampler Ultrafiltration Cartridge, UFP‐100C‐4×2MA (Cytiva, USA), were used for sIONP purification. Dilution to a concentration of 0.72 mg Fe mL^−1^ was used for purification with the parameters listed in Table S1 (Supporting Information). The sIONP solution underwent diafiltration, during which the retentate was circulated through the cartridge and the feed reservoir at a flow rate of 300 mL min^−1^ with a continuous supply of ultra‐pure water (12 L) from the external reservoir at a flow rate of 120 mL min^−1^, while maintaining a constant volume of 400 mL. At the end, the system was set into concentration mode, during which the retentate was concentrated, collected, and characterized 2 h 30 min after the start of purification. The filtrates were carefully discarded, and the TFF system was cleaned and stored in 20% ethanol.

### Characterization of Purified sIONP

sIONP purified via both ultracentrifugation and tangential flow filtration (TFF) were characterized using dynamic light scattering (DLS) and zeta potential measurements performed on a Brookhaven Zeta PALS instrument (Brookhaven Instruments Corporation) equipped with a 635 nm diode laser at 15 mW power. Transmission electron microscopy (TEM) analysis was conducted using a Tecnai T12 microscope (FEI, OR) operating at 120 kV, with samples prepared on carbon film 200‐mesh copper grids. Iron content was quantified by inductively coupled plasma optical emission spectrometry (ICP‐OES) using a Thermo Fisher Scientific iCAP 6500 dual‐view instrument operating at 1150 W.

### High‐Resolution Magic‐Angle‐Spinning NMR measurement

The impact of the diafiltration mechanism of the TFF system on the sIONP coating was quantified by high‐resolution magic‐angle‐spinning (HRMAS) NMR measurement using our previous method.^[^ ^19^ ^]^ After purification, ultracentrifuged, and TFF‐purified sIONP were lyophilized, added to 99.9% D_2_O, and bath sonicated for 30 min to disperse the sIONP completely, then 10 µL containing 0.5 mgFe mL^−1^ was loaded into each tube insert of the HRMAS NMR, and pure solvent (20 µL) was added. The insert was sealed with an inner pressure cap followed by a secondary outer screw cap and a disposable HRMAS insert kit. The insert was thereafter placed into a zirconium tube and pressure‐capped using a 4 mm MAS Bruker rotor kit. Immediately, zirconium tubes containing samples were loaded onto a Bruker 700 MHz Avance NMR system equipped with a 4 mm HRMAS inverse detection probe and spun at 6000 Hz at 25 °C, D_2_O was locked, and the probe was tuned for ^1^H acquisition. Manual shim adjustments were performed using a single‐scan acquisition with a 0.5 s refresh interval. The peak shape and full width at half maximum (FWHM) of the D_2_O signal were optimized by adjusting shim parameters: Z, Z^2^, ZY, Y, ZY^2^, X, Z^4^, and Z^5^. Peaks from the D_2_O were manually shimmed for best peak resolution, where the highest‐grade D_2_O gave the best results. Easy data processing and increased resolution were achieved by phase spectra adjustment during shimming.

### Stability Studies

CPA solutions VMP (12.9% formamide, 16.8 wt% ethylene glycol, 1% X‐1000, 22.3% DMSO, 1% Z‐1000) and VS55 (24.2% DMSO, 16.8 wt% propylene glycol, 0.24% HEPES, 14.0% formamide) were formulated and maintained at 4 °C before stability studies. EMG 308, ultracentrifuged sIONP, and TFF‐purified sIONP were mixed thoroughly into the CPAs at an iron concentration of 1 mg mL^−1^. Samples were maintained at 23 ± 2 °C for several months, and DLS measurements were performed over time to determine the sizes of the sIONP.

### Specific Absorption Rate (SAR)

A 360 kW HotShot inductive heating system equipped with a 2.75‐turn, water‐cooled copper coil (Ameritherm Inc., Scottsville, NY) was usedat 20 kAm^−1^ and 360 kHz for SAR measurement. 1 mL (4 mgFe mL^−1^) each of each; EMG 308, ultracentrifuged sIONP, and TFF‐purified sIONP were added to a cryotube sealed with a rubber stopper, a fiber‐optic thermocouple was inserted through the center, and temperature measurements were recorded for 120 s at 1 s intervals. Temperature stabilization was done for 30 s, 60 s during coil activation, and 30 s after the coil was turned off. SAR calculation was carried out with the first 30 seconds of heating data using the equation below, with water as the reference sample.^[^ ^15^ ^]^

(1) $$\mathrm{SAR}=\frac{\rho VC_{\mathrm{water}}}{\mathrm{Fe}\;g}\left(\frac{\Delta T_{\mathrm{IONP}}}{\Delta t_{IONP}}-\frac{\Delta T_{\mathrm{water}}}{\Delta t_{\mathrm{water}}}\right)$$

### Raman Spectroscopy

LabRAM by Horiba HR Evolution confocal Raman with an excitation wavelength of 532 nm laser at 50x amplification was used to obtain the Raman spectra. EMG 308, ultracentrifuged sIONP, and TFF‐purified sIONP in water were freeze–dried under −50 °C and high vacuum to avoid oxidation. Samples were transferred onto a glass slide and sealed with coverslips and tape, and spectra values were acquired using five accumulations for 10 s, a laser intensity of 10%, a pinhole of 200 µm, and a grafting of 600 (500 nm).^[^ ^32^, ^41^ ^]^

### Cytotoxicity of sIONP on Human Dermal Fibroblast Cells (HDF)

Human dermal fibroblast (HDF, ATCC's PCS‐201‐012) cells were cultured in Eppendorf cell culture dishes containing Dulbecco's Modified Eagle Medium (DMEM) supplemented with 10% fetal bovine serum (FBS) (Gibco, life technologies), and 1% penicillin‐streptomycin (Sigma), and maintained at 37 °C in a humidified incubator with 5% CO_2_ as previously reported.^[^ ^20^ ^]^ The cells were seeded into a 96‐well microplate (Thermo Fisher Scientific), grown for 24 h, and thereafter treated with EMG 308, ultracentrifuged sIONP, and TFF‐purified sIONP at a concentration of 10 mgFe mL^−1^, followed by incubation for 24 h at 37 °C in 5% CO_2._ The cells were washed five times with Hank's Balanced Salt Solution (HBSS) to thoroughly remove unbound IONP, and cell viability was determined using the Hoechst‐PI assay.

### Kidney Recovery and Cannulation for Perfusion and Transplantation

Donor rats were anesthetized using isoflurane, followed by a cruciate laparotomy. The bowel and mesentery were retracted to expose the abdominal vasculature. The abdominal aorta, inferior vena cava, and left renal artery and vein were carefully mobilized for cannulation. The kidney was freed from Gerota's fascia, and a 20G bulb‐tip catheter (FTP‐20‐30, Instech Laboratories, Plymouth Meeting, PA) was inserted into the abdominal aorta just below the renal arteries. For venous drainage during ex vivo perfusion (but not during transplantation procedures), the inferior vena cava was cannulated using a Male Luer to Hose Barb Adapter (45518‐46, Cole Parmer, Vernon Hills, IL). Additionally, the ureter was cannulated using PE‐10‐100 polyethylene tubing (0.011″ ID × 0.025″ OD; SAI Infusion Technologies). The suprarenal aorta was then cross‐clamped, and the kidney was perfused with 15 mL of cold University of Wisconsin (UW) solution (0°C–4 °C) containing 500 IU of heparin. Subsequently, the suprarenal aorta and vena cava were ligated and transected above the renal vessels. The kidney was then explanted and preserved in UW solution at 4 °C until it was subjected to hypothermic machine perfusion.^[^ ^3^ ^]^

### Perfusion CPA Loading into the Kidneys

A modified VMP loading protocol and a customized multi‐thermic perfusion system were used for pressure‐ or flow‐regulated perfusion.^[^ ^3^, ^16^, ^18^ ^]^ VMP (1% Z‐1000,16.8 wt% ethylene glycol, 1% X‐1000, 22.3% DMSO, 12.9% formamide) prepared in a carrier solution (LM5‐XZ) was used for CPA loading. First, the kidney was flushed 20 min with a modified carrier solution (LM5‐XZ) supplemented with synthetic ice blockers X‐1000 and Z‐1000,^[^ ^6^ ^]^ aiming to enhance vascular perfusion through its oncotic properties. Full‐strength CPA (VMP) was gradually reached by initially ramping the concentration from 0 to 5 m at 50 mM min^−1^, held for 10 min, and then stepped up to 8.4 m (full‐strength VMP) to reduce exposure time at the highest CPA concentration. A reduction in flow rate was associated with an increase in viscosity. To maintain adequate flow rates during the 25 min perfusion with full‐strength VMP, the perfusion pressure was elevated from 40 to 60 mmHg. The total duration of VMP perfusion loading into the kidney was 155 min.^[^ ^3^ ^]^

### sIONP Loading into the Kidneys

Immediately after the last step of VMP loading, ultracentrifuged sIONP and TFF‐purified sIONP at 10 mgFe mL^−1^ concentration in VMP were perfused into the kidney via a syringe pump at 4 °C, at a steady flow rate of 0.5 mL min^−1^, maintained for 4.5 min, and the corresponding loading pressure was documented.^[^ ^18^ ^]^ The TFF‐sIONP loaded kidneys were compared with ultracentrifuged sIONP loaded kidneys treated with the same protocol and published in.^[^ ^3^ ^]^

### Vitrification of sIONP Loaded Kidneys

A cryogenic 2 × 3 inch polyethylene zipper‐lock bag (McMaster‐Carr, Elmhurst, IL) was preloaded with 15 mL of ultracentrifuged or TFF‐purified sIONP (4 mgFe mL^−1^) in 100% VMP and stored in a refrigerator at 4 °C. After the sIONP loading step, the kidneys were gently disconnected from the perfusion system, and a cryogenic temperature‐sensing fiber optic probe (Quanlitrol, Fairport, NY) was inserted in the cryobag near to the kidney and held with cello tape. The kidneys were gently placed in the center of a Kryo 560‐16 device (Planer Ltd, Middlesex, UK) controlled‐rate freezer for vitrification. The Kryo 560‐16 device controlled‐rate freezer was preprogrammed to start at 0 °C (Tchamber temperature) and cool down to −122 °C at a ramp rate of −40 °C min^−1^. When the chamber reached −122 °C, a 25 min annealing phase was introduced to allow thermal equilibration of the organ and minimize the introduction of thermal stress before the glass transition phase. At the end of the annealing step, a ramp rate of −5 °C min^−1^ was used to further cool the organ from −122 to −150 °C, further reducing thermal stress during the glassy phase. When the chamber temperature reached −150 °C, the temperature was held for 10 min to stabilize the organ to the storage temperature (−150 °C). Then, the organ was swiftly transferred to a −150 °C freezer (PHC Corporation of North America, Wood Dale, IL) for storage before rewarming.^[^ ^3^ ^]^ The TFF‐sIONP vitrified kidneys were compared with ultracentrifuged sIONP vitrified kidneys treated with the same protocol and published in.^[^ ^3^ ^]^

### Nanowarming of vitrified kidneys

Nanowarming was performed using a radiofrequency (RF) coil (AMF Life Systems, Auburn Hills, Michigan) operating at 94% power, generating an RF field of 63 kA m^−1^ at 180 kHz, with field variation maintained within ±5% across the ≈80 mL coil bore. The vitrified kidney, previously stored at −150 °C, was promptly placed at the center of the RF coil, and nanowarming was initiated by activating the alternating magnetic field. The thermal heating was logged until the temperature reached −25 °C (which is above the VMP melting temperature). The field was switched off, and the cryobag holding the kidney was removed from the coil and immediately placed on ice. Unloading the VMP and sIONP started immediately using the perfusion system.^[^ ^3^ ^]^ The TFF‐sIONP nanowarmed kidneys were compared with ultracentrifuged sIONP nanowarmed kidneys treated with the same protocol and published in.^[^ ^3^ ^]^

### Unloading of CPA and sIONP From Nanowarmed Kidney

The kidney samples were reconnected to the perfusion system, and 4.2 m VMP with 300 mm mannitol was perfused through the kidney for 15 min. Subsequently, the 4.2 m VMP solution containing 300 mm mannitol was gradually ramped up to 0 m (LM5‐X2) over a 120 min ramp‐down period. Mannitol and VMP ramping rates were −2.5 and −35 mM min^−1^, respectively. Then, the kidney was perfused with LM5‐XZ for 30 min. The unloading pressure was maintained at 40 mmHg and the temperature between 0 and 4 °C for the total perfusion time of 165 min. The kidney was flushed with cold University of Wisconsin (UW) hypothermic preservative solution, carefully disconnected from the perfusion system, and returned to UW solution on ice.^[^ ^3^ ^]^ The TFF‐sIONP unloaded kidneys were compared with ultracentrifuged sIONP unloaded kidneys treated with the same protocol and published in.^[^ ^3^ ^]^

### Micro‐CT Imaging of Vitrified Kidney

Micro‐CT imaging at an accelerating voltage of 65 kV and current of 95 µA was performed at 0.061 mm resolution using an imaging system (Nikon XTH 225, Nikon Metrology Melville). During imaging, the cryobag containing the vitrified kidney was maintained in liquid nitrogen vapor (−150 °C) within a Styrofoam container. Separate tubes containing water and air at room temperature were positioned at the top of the container to serve as calibration references for determining radiodensity in Hounsfield units (HU). Beam‐hardening artifacts were reduced, and image quality improved (3D CT pro, Nikon Metrology, Mu) by reconstruction, and the image conversion to a 16‐bit float image using unsigned integers, post‐processed (VG studio Max 3.2, volume Graphic, NC) was carried out. For final analysis, the images were exported as DICOM images using MATLAB (MathWorks).^[^ ^3^, ^37^ ^]^

### Histology

Kidneys recovered for histological analysis were bisected and preserved in 4% paraformaldehyde until they were processed for paraffin embedding and bench‐top sectioning. Histology with hematoxylin and eosin (H&E) was performed as previously reported.^[^ ^18^ ^]^ The kidney slices were digitized for histopathological analysis and interpreted following standard protocols. Kidneys were divided into four sections to assess the remnant particles after perfusion in the cortex, cortico‐medullary junction, medulla, and pelvis. Particles were characterized into ≤10 µm, 10–50 µm, and >50 µm. A two‐tailed *t*‐test was conducted to assess statistical significance, with a *p *< 0.05 considered indicative of significance.

### Post‐Perfusion Iron Quantification in the Kidney

Iron quantification of control kidneys, ultracentrifuge, and TFF‐purified sIONP loaded, vitrified, nanowarmed, and unloaded kidneys was conducted using Inductively Coupled Plasma Mass Spectrometry (ICP‐MS) by ALS Global with an Agilent 7700. Following sIONP unloading from the kidneys, the tissues were dried overnight in a vacuum oven at 120°C and then finely powdered. An aliquot of each powdered sample was placed into a Teflon digestion vessel with 15% concentrated nitric acid and 5% concentrated hydrochloric acid. The vessel was sealed and incubated in a 105 °C oven for at least 12 h. After cooling, the digested samples were transferred to centrifuge tubes and diluted with deionized water to a final volume of 20 mL.^[^ ^42^ ^]^

### Transplantation Studies

Inbred male Lewis rats (450–500 g) were used as both donors and recipients for syngeneic transplants. Baseline serum creatinine levels were measured before and after kidney transplantation. Donor surgery is described above.

Recipient rats were anesthetized with isoflurane, followed by a laparotomy. The bowel and mesentery were carefully mobilized and retracted. The abdominal aorta, inferior vena cava, and left renal artery and vein were dissected free from surrounding tissue. The left native kidney was freed from Gerota's fascia and skeletonized down to the hilar vessels. The left native ureter was transected near the hilum to preserve maximum length. The left renal artery was then ligated and divided. A microvascular clamp was applied to the renal vein, which was subsequently divided to remove the native kidney.

Two arterial microvascular clamps were placed on the recipient's infrarenal aorta—one proximal and one distal to the planned anastomosis site. An arteriotomy was created and immediately flushed with heparinized saline. The donor kidney was flushed with cold normal saline, wrapped in cold gauze, and lowered into the surgical field, with cold saline periodically dripped onto it to maintain hypothermia until reperfusion. An end‐to‐side anastomosis was performed between the donor renal artery and the recipient's aorta using running 10–0 Prolene sutures. Next, an end‐to‐side venous anastomosis was completed between the donor renal vein and the inferior vena cava. After both arterial and venous anastomoses were secured, the clamps were released to allow reperfusion of the kidney. Warm normal saline was then poured over the reperfused kidney. After urine was observed in the catheter, the native renal arteries, veins, and ureters were ligated and divided, and the kidneys were explanted. An end‐to‐end anastomosis of the donor and recipient ureters was performed over a PE‐10 stent. The abdomen was then closed in layers using 4–0 PDS sutures for the abdominal wall and 5–0 PDS sutures for the skin. The rat was placed on a heating pad and given supplemental oxygen until it fully recovered from anesthesia. The rats were euthanized on postoperative day 26, and the kidneys were collected for histological analysis. The TFF‐sIONP kidneys were compared with ultracentrifuged sIONP kidneys treated with the same protocol and published in.^[^ ^3^ ^]^

### Statistical Analysis

Histological analysis was performed using Proscia, and statistical analyses were conducted using GraphPad Prism version 10.2.0. Normality of continuous variables was assessed using the Shapiro–Wilk test or q‐q plots, and frequency distribution histograms. Data are presented as mean ± standard deviation (SD), and each figure legend includes the number of replicates and the specific statistical methods used. Homogeneity of variance was evaluated using the F‐test, Bartlett's test, or Brown–Forsythe test. For comparisons between normally distributed groups, a two‐tailed *t*‐test was used for single comparisons, and ANOVA followed by Tukey's Honestly Significant Difference (HSD) post hoc test was used for multiple comparisons with equal variances. All statistical tests were two‐sided, and a *p* < 0.05 was considered statistically significant. Where applicable, *p* values were adjusted for multiple comparisons.

## Conflict of Interest

There are patents on the nanowarming technique. Some companies have indicated interested in licensing the technology.

## Supporting information



Supporting Information

## Data Availability

All raw data have been archived for public access at DRUM: https://hdl.handle.net/11299/271383
