# Supplementary material for: Scalable Purification of Iron Oxide Nanoparticles for Organ Cryopreservation and Transplantation
Source: Small. 2025 Sep 24;21(44):e04910. doi: 10.1002/smll.202504910 (PMC12590514; doi:10.1002/smll.202504910)
Supplement: Supplementary file 1 — Supporting Information [file SMLL-21-e04910-s001.docx]

Supplementary information

**Scalable Purification of Iron Oxide Nanoparticles for Organ Cryopreservation and Transplantation**

Onyinyechukwu Justina Oziri^1^, Joseph Sushil Rao^2^, Cameron Scheithauer^1^, Zonghu Han^1^, Saurin Kantesaria^3,5^, Diane Tobolt^2^, Haopu Liang^4^, Michael L Etheridge^1^, Yadong Yin^4^, Erik B Finger^2^*, John C Bischof^1,5,6^*

^1^ Department of Mechanical Engineering, University of Minnesota, Minneapolis, MN, 55455, USA

^2^ Department of Surgery, University of Minnesota, Minneapolis, MN, 55455, USA

^3^ Center for Magnetic Resonance Research and Department of Radiology, University of Minnesota Medical School, MN, 55455, USA

^4^Department of Chemistry, University of California, Riverside, CA, 92512, USA

^5^ Department of Biomedical Engineering, University of Minnesota, Minneapolis, MN, 55455, USA

^6^ Institute for Engineering in Medicine, University of Minnesota, Minneapolis, MN, 55455, USA

* Corresponding authors

**Table S1**. TFF parameters used for purification of sIONPs

| Parameters | TFF | Ultracentrifugation |
| --- | --- | --- |
| sIONPs loading concentration per ml | 0.72 mg Fe/ml | 2.4 mg Fe/ml |
| sIONPs concentration purified * | 0.72 g Fe | 0.72 g Fe |
| Rentante flow rate (ml/min) | 300 ml/min | - |
| Transfer flow rate (ml/min) | 120 ml/min | - |
| Transmembrane pressure TMP (psi) | 8.0-6.0 | - |
| Ultra-pure water (L) | 12 L | 630 mL |
| Purification time | 2 hours 30 minutes | 3 days |
| Ethanol | 0 mL | 2 L |

- NOTE: Our silica coating capability is 1.44 g Fe[1]. This was split into two 0.72 g Fe in order to directly compare ultracentrifugation and TFF for purification.


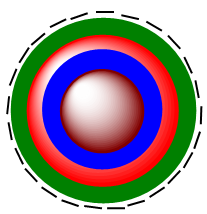

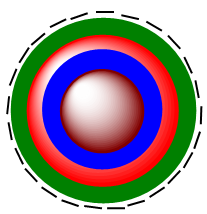


1. Ultracentrifuged sIONP
2. TFF-purified sIONP
3. EMG 308

93 ± 4 nm


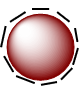


88 ± 5 nm

73 ± 3 nm

**Figure S1**. **Schematic illustrations of** (a) EMG 308, (b) Ultracentrifuged sIONP (c) TFF purified-sIONP sizes determined by DLS. Blue coating represents silica, red represent PVP, and green represent PEG. Data represents mean ± SD. n = 4 replicates per group. Statistical comparison of DLS size between ultracentrifuged and TFF-purified sIONP using two-tailed t-test is not significant (p = 0.210)

**Table S2.** ***ζ*-Potential values of sIONPs by different purification**

|  | EMG 308 | Ultracentrifuged sIONP | TFF-purified sIONP |
| --- | --- | --- | --- |
| *ζ*-Potential | -50 ± 1 mV | -37 ± 1 mV | -41± 2 mV |

1. TFF-purified sIONPs
2. Ultracentrifuged sIONPs


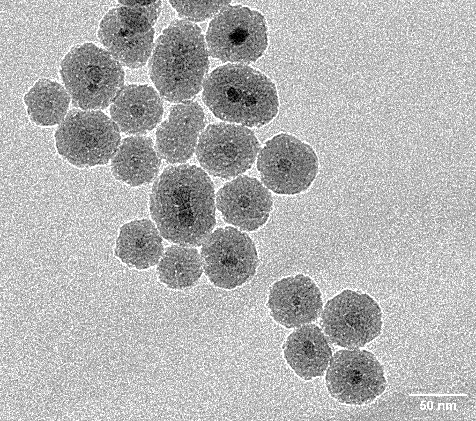

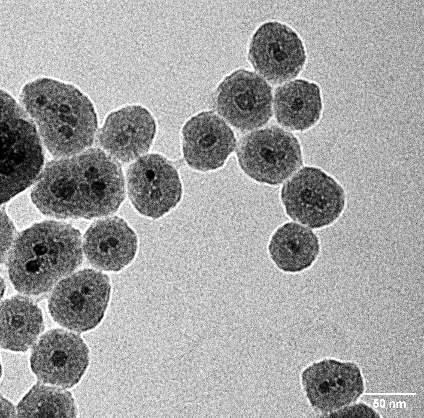


**Figure S2. TEM sizes of** (a) Ultracentrifuged sIONP (n = 6, 49 ± 1 nm) (b) TFF-purified sIONP (n = 10, 47 ± 3 nm). Data represents mean ± SD. Statistical comparison of TEM size between ultracentrifuged and TFF-purified sIONP using two-tailed t-test is not significant (p =0.244).


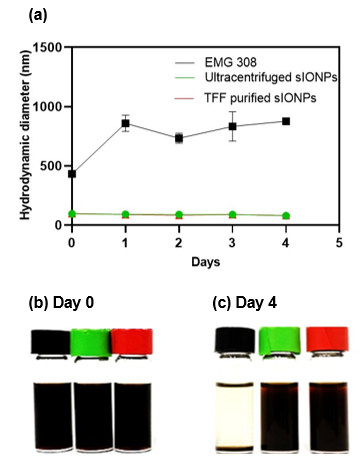


**Figure S3. Stability test of EMG 308 and sIONP in VS55 CPA.** (a) DLS sizes (n = 3) of EMG 308, ultracentrifuged sIONP and TFF-purified sIONP after 4 days. Photographs of EMG 308 (black), ultracentrifuged sIONP (green), and TFF-purified sIONP (red) (a) immediately after and (b) 4 days after the addition to VS55 solution. Data represents mean ± SD. n = 3 replicates per group. Statistical comparison between ultracentrifuged and TFF-purified sIONP on day 4 using two-tailed t-test. ns, not significant

**Figure S4**. HRMAS measurement of PEG signals from ultracentrifuged sIONP (green) and TFF-purified sIONP (red).


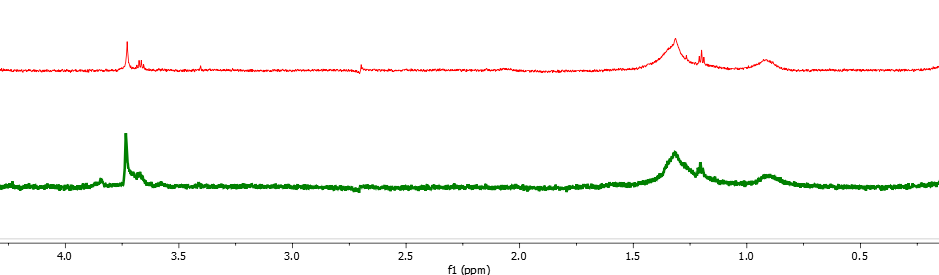


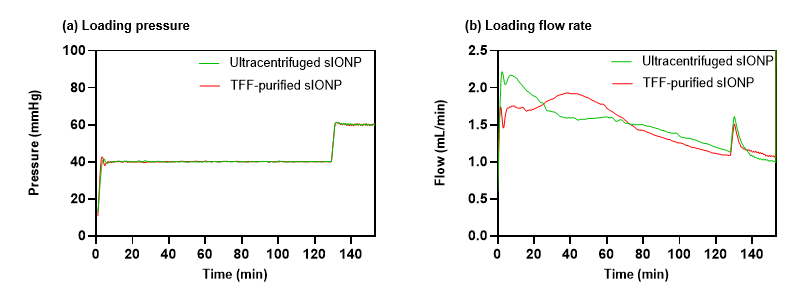


**Figure S5**. **Example perfusion loading pressure** (a), and arterial flow rate (b), of ultracentrifuged and TFF-purified sIONP in rat kidneys. The TFF-sIONP loaded kidneys were compared with ultracentrifuged sIONP loaded kidneys treated with same protocol and published in [3]


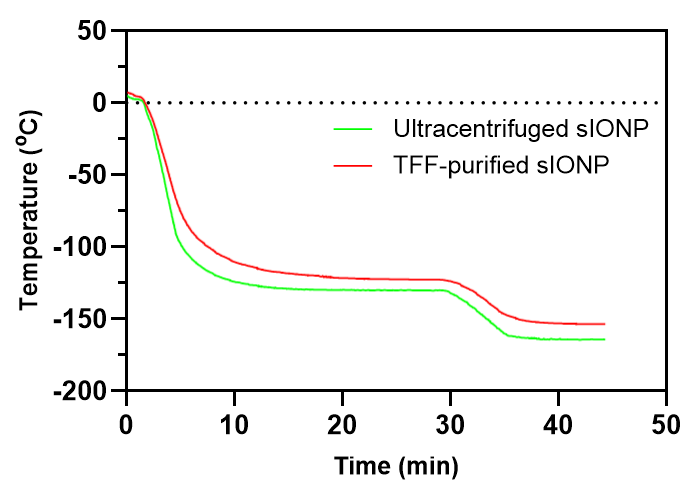


**Figure S6**. **Example thermal profile of sIONP during vitrification**. Both ultracentrifuged and TFF-purified sIONP in CPA were cooled from above 0 *°*C to an annealing step just above the glass transition temperature (*T*_g_ = −128.3 °C), and finally slower cooling into the glassy phase (annealing). The cooling rates of TFF-sIONP kidneys were compared with those of ultracentrifuged sIONP kidneys treated using the same protocol, as reported in [3].


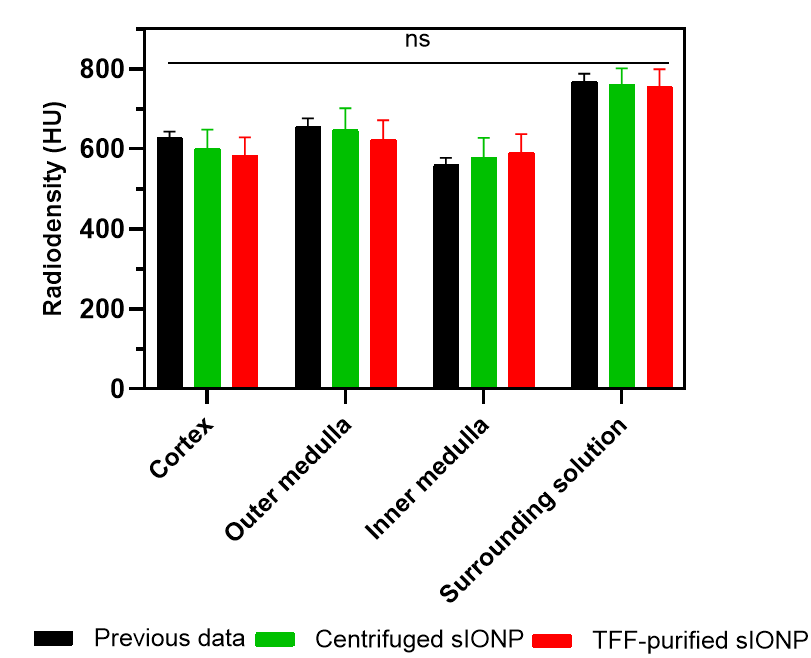


**Figure S7**. **Radiodensity data represented in Hounsfield units (HU) of vitrified kidneys.** The HU scale is used to differentiate between frozen and vitrified kidneys based on attenuation. A low attenuation of ˂ 400 HU is indicative of a frozen kidney, while > 500 HU indicates a vitrified kidney[2, 3]. Statistical comparison showed no significant differences (n = 3, P < 0.05) in the various regions of the kidney between ultracentrifuged and TFF-purified sIONP-loaded kidney (cortex; p = 0.669, outer medullar; p = 0.620, inner medullar; p = 0.795, and surrounding solution; p = 0.920) , ultracentrifuged sIONP loaded kidneys and previous data treated with same protocol and published in [3] (cortex; p = 0.629, outer medullar; p = 0.941, inner medullar; p = 0.812, and surrounding solution; p = 0.955), or TFF purified sIONP-loaded kidney and previous data[3] (cortex; p = 0.313, outer medullar; p = 0.523, inner medullar; p = 0.559, and surrounding solution; p = 0.907). Data represents mean ± SD. One-way ANOVA followed by Tukey’s post hoc test was used for statistical comparison. ns; not significant at p < 0.05.


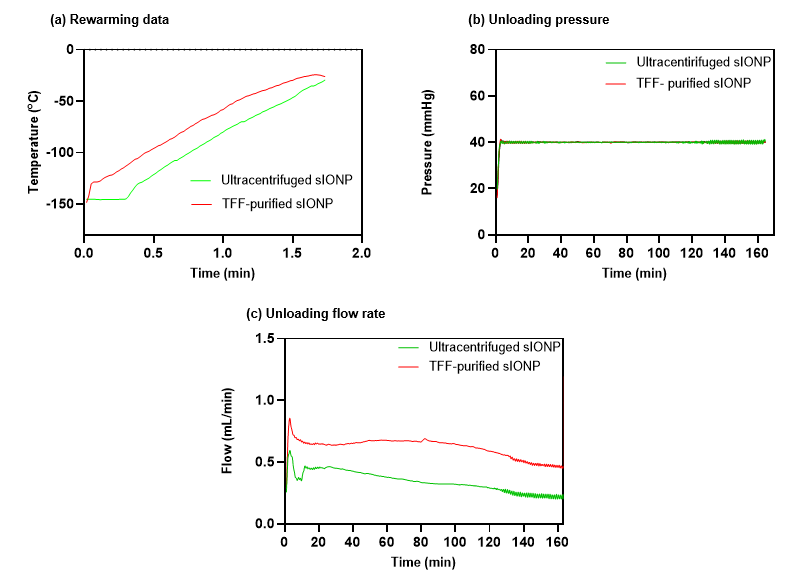


**Figure S8. Rewarming and perfusion unloading data for sIONP CPA solutions.** (a) Example rewarming thermal curve, (b) perfusion unloading pressure and, (c) arterial flow rate in rat kidneys. The rewarming rate, pressure, and flow rate of TFF-sIONP kidneys were compared with those of ultracentrifuged sIONP kidneys treated using the same protocol, as reported in [3].

**
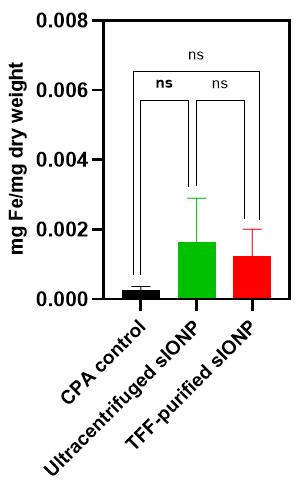
**

**Figure S9. ICP Mass Spec measurement of Residual Fe concentration after unloading of the control, ultracentrifuged (n = 3) and TFF-sIONP purified kidneys (n = 4).** Statistical comparison showed no significant differences between ultracentrifuged and TFF-purified sIONP, control and ultracentrifuged sIONP, or control and TFF-purified sIONP. Data represents mean ± SD. One-way ANOVA followed by Tukey’s post hoc test was used for statistical comparison. ns; not significant at p < 0.05


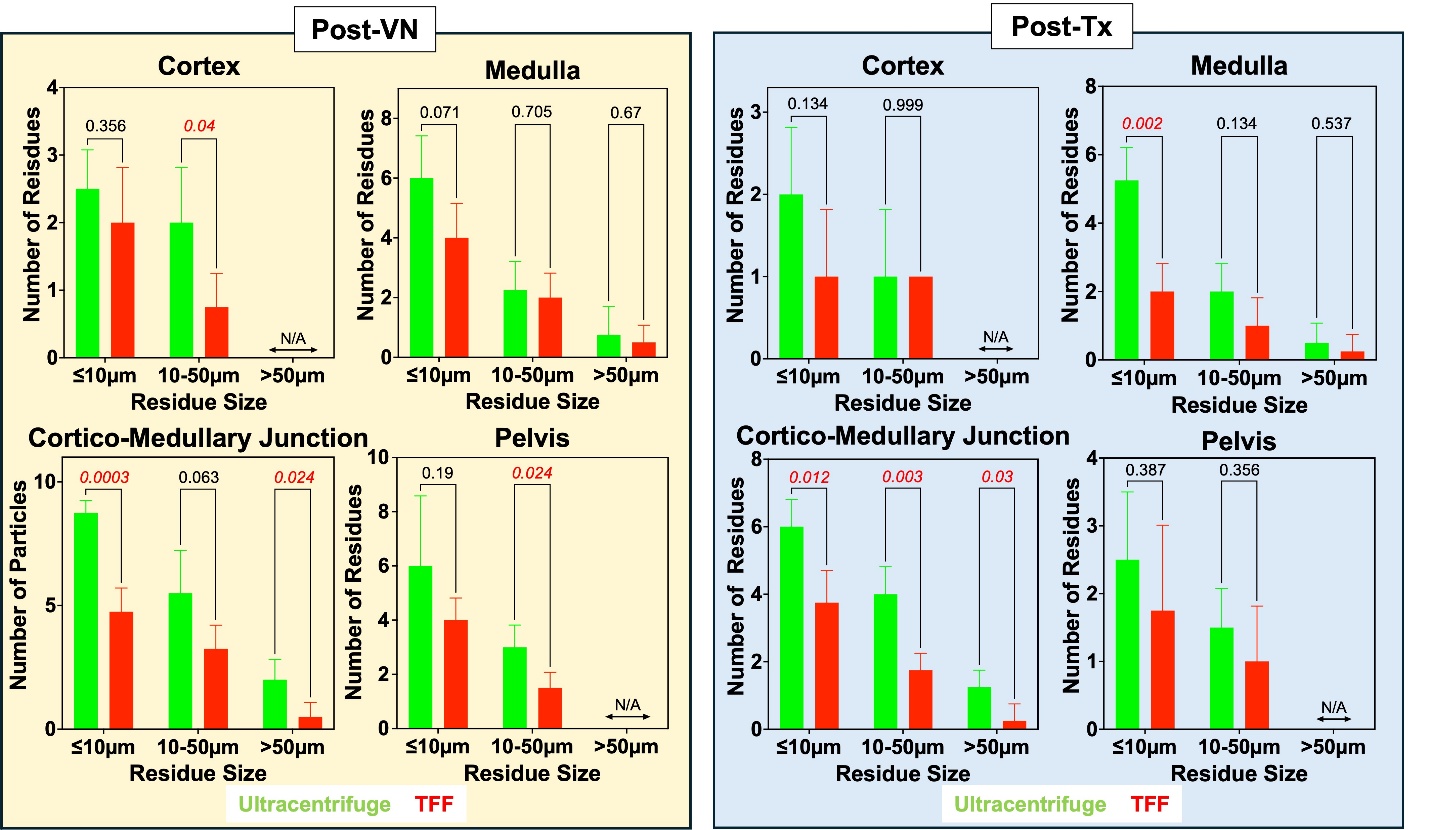


**Figure S10**. **Residue distribution per region of vitrified and nanowarmed kidney following CPA-sIONP unloading.** Number of brown residues (N) per field of view (n=4) in the cortex, cortico-medullary junction, medulla and the pelvis in the TFF and ultracentrifuge groups reported in [3]. Data represents mean ± SD. Two-tailed t-test was used for statistical comparison. n = 4 replicates per group, *p < 0.05, **p < 0.01, ***p < 0.001.


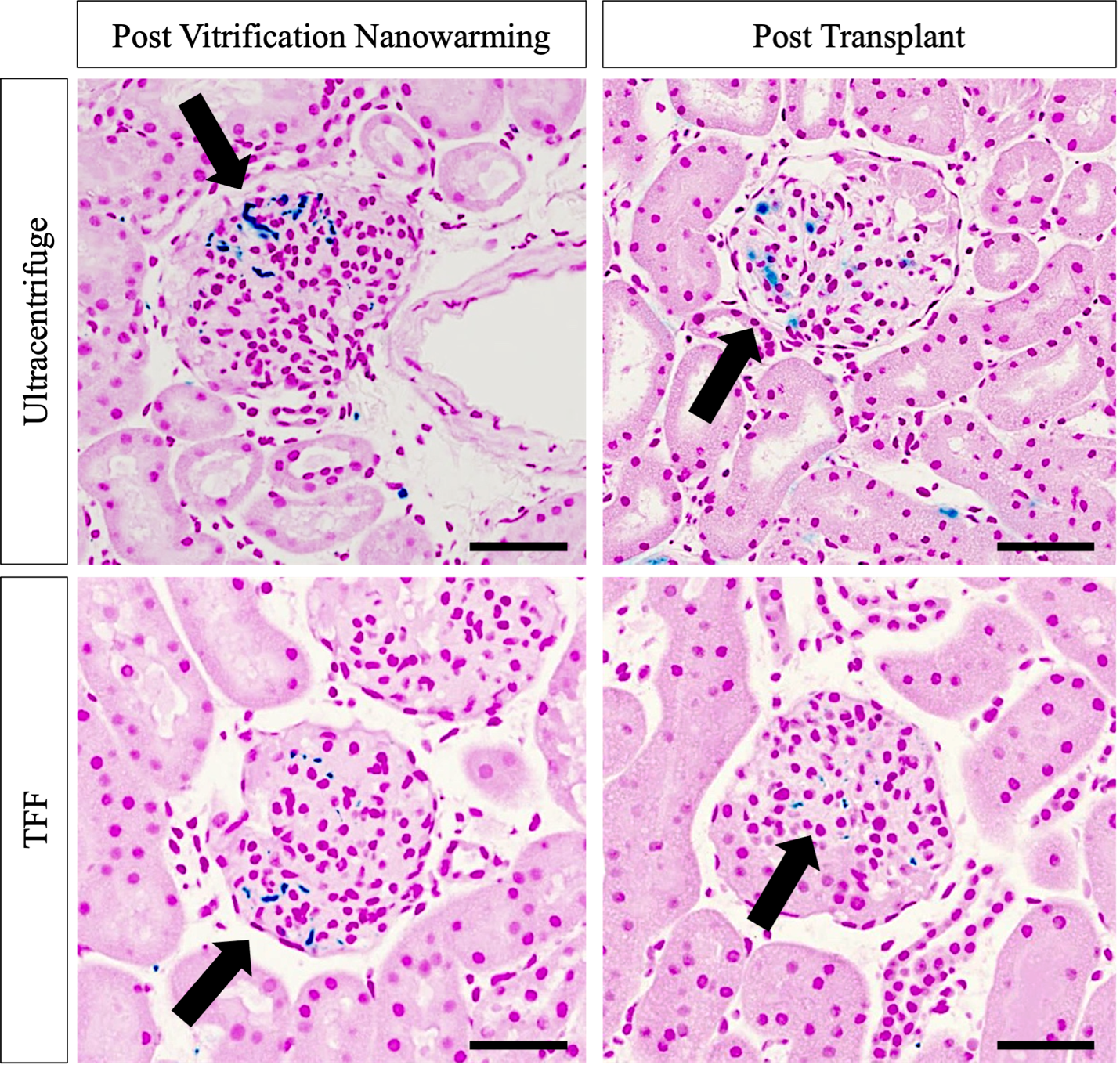


**Figure S11. Rat kidneys following vitrification and rewarming using sIONPs purified by either ultracentrifugation or TFF were stained with Prussian blue**. The staining highlights the presence of residual sIONP within the glomeruli. TFF-sIONP kidneys were compared with those of ultracentrifuged sIONP kidneys treated using the same protocol, as reported in [3].

**Table S3. Batch variation in DLS sizes of sIONPs purified by different purification**

| Batch number | 1 | | 2 | | 3 | |
| --- | --- | --- | --- | --- | --- | --- |
| Purification method | UC | TFF | UC | TFF | UC | TFF |
| DLS size (nm) | 145 ± 2 | 86 ± 3 | 121 ± 2 | 81 ± 2 | 94 ± 3 | 91 ± 2 |

**Reference**

[1] Z. Gao *et al.*, "Preparation of Scalable Silica-Coated Iron Oxide Nanoparticles for Nanowarming," *Advanced Science,* vol. 7, no. 4, Feb 2020, Art no. 1901624, doi: 10.1002/advs.201901624.

[2] Z. H. Han *et al.*, "Diffusion Limited Cryopreservation of Tissue with Radiofrequency Heated Metal Forms," *Advanced Healthcare Materials,* vol. 9, no. 19, Oct 2020, Art no. 2000796, doi: 10.1002/adhm.202000796.

[3] Z. H. Han *et al.*, "Vitrification and nanowarming enable long-term organ cryopreservation and life-sustaining kidney transplantation in a rat model," *Nature Communications,* vol. 14, no. 1, Jun 2023, Art no. 3407, doi: 10.1038/s41467-023-38824-8.
